# Silent Intruders: The Gut Virome in Brain Aging and Cognitive Decline

**DOI:** 10.3390/pathogens15020180

**Published:** 2026-02-06

**Authors:** Serena Silvestro, Angelina Midiri, Carmelo Biondo, Selene Casilli, Lucia Borrello, Sebastiana Zummo, Giuseppe Mancuso

**Affiliations:** 1 IRCCS Centro Neurolesi “Bonino-Pulejo”, Via Provinciale Palermo, Contrada Casazza, 98124 Messina, Italy; serena.silvestro@irccsme.it; 2 Mycology Laboratory, Department of Human Pathology, University of Messina, 98125 Messina, Italy; amidiri@unime.it (A.M.); cbiondo@unime.it (C.B.); selenecasilli@gmail.com (S.C.); lucia.borrello94@gmail.com (L.B.); sebastiana.zummo@unime.it (S.Z.)

**Keywords:** gut virome, brain aging, cognitive decline, neuroinflammation, neurodegenerative disease

## Abstract

Recent advances in next-generation sequencing have revealed that the virome—the set of viruses residing in the gastrointestinal tract—is a fundamental yet still underexplored component of the human intestinal ecosystem. Despite the prevalence of research focused on bacterial alterations, recent findings suggest a significant role for viral elements within the intestinal microbiota, namely latent viruses, bacteriophages and eukaryotic viruses, in influencing brain health. Alterations in the gut virome may, in particular, contribute to the processes underlying brain aging, cognitive decline, and neurodegenerative diseases such as Alzheimer’s, Parkinson’s, and multiple sclerosis (MS). This review highlights the potential of intestinal viruses to modulate gut barrier integrity, systemic immune response and neuroimmune inflammation. Such interactions could promote conditions of chronic neuroinflammation, alterations in synaptic plasticity, and neuronal vulnerability. A more comprehensive understanding of the role of the gut virome could potentially result in novel approaches to the early detection and treatment of neurocognitive disorders in adults and older individuals.

## 1. Introduction

Neurodegenerative diseases represent a group of chronic and progressive conditions characterized by the selective and progressive loss of neurons in specific brain regions, causing severe cognitive and/or motor decline [1]. Among these diseases, Alzheimer’s disease (AD) and Parkinson’s disease (PD) are the most prominent [2,3]. Given their increasing global incidence and rising life expectancy, they represent one of the most urgent medical challenges of our time [4].

Despite their high heterogeneity, neurodegenerative diseases share fundamental pathogenic mechanisms, including the accumulation and aggregation of misfolded proteins (such as β-amyloid (Aβ) and tau protein in AD or alpha-synuclein (α-syn) in PD) and persistent neuroinflammation [5,6]. Their etiology is complex and multifactorial, closely linked to age and resulting from a combination of genetic and environmental factors. In contrast to traditional research, which concentrated exclusively on the central nervous system (CNS), contemporary research has shifted its focus to the pivotal bidirectional interaction between the brain and the gastrointestinal tract, known as the “gut–brain axis” [7,8].

The preclinical and clinical evidence indicates that alterations in the composition and function of the gut microbiota and virome (the viral component, including bacteriophages) are not merely a consequence of these conditions, but they also represent a potential causal and key modulating factor in neuroinflammation and the progression of these conditions [9,10,11].

In this context, the gut microbiota and its viral component, the virome, have emerged as key players. To understand the role of these biological entities, it is essential to distinguish between the terms microbiota and microbiome. The gut microbiota refers to the diverse community of microorganisms—including bacteria, archaea, fungi, and viruses—that inhabit the gastrointestinal tract. While the term “microbiota” in the scientific literature has been historically dominated by the study of bacteria, it is crucial to recognize that viruses (infecting both bacteria and eukaryotic host cells) constitute a massive and integral part of this ecosystem. In contrast, the microbiome is a broader term encompassing the entire habitat, including the microorganisms, their genomes (genes), their metabolites, and the surrounding environmental conditions [12]. A critical yet less explored component of this complex environment is the gut virome, defined as the collective assembly of all viral elements in the gut, comprising bacteriophages, eukaryotic viruses, and virus-derived genetic elements [13]. Consistent with this definition, alterations in the microbiota (dysbiosis) have been widely documented in patients with AD and PD with distinct microbial profiles modulating inflammation and the production of neurotransmitters and metabolites [8]. In particular, while the bacterial component has been the primary focus of research, it is now clear that the gut virome consists mainly of bacteriophages (or phages), which specifically infect bacteria and not human cells directly. The main mechanism by which the virome influences neurodegeneration is indirect, through it has a dynamic interaction with the bacterial microbiota, inducing the lysis of beneficial bacterial species such as those that produce short-chain fatty acids (SCFAs) or neurotransmitters such as gamma-aminobutyric acid (GABA) [14]. The lysis of beneficial bacterial species causes the release of bacterial components (such as lipopolysaccharides, LPSs) into the intestinal environment, causing peripheral inflammation and increased intestinal permeability (“leaky gut”) [15]. Furthermore, the expansion of pathobionts in AD and PD might be facilitated by prophage induction. Stress conditions in the neurodegenerative gut can trigger the transition of lysogenic phages to the lytic cycle, which not only alters the bacterial community structure but also releases pathogen-associated molecular patterns (PAMPs) that exacerbate neuroinflammation [9]. Increased intestinal permeability allows toxins and inflammatory cytokines to enter the systemic circulation, cross the compromised blood–brain barrier (BBB), and activate resident immune cells in the brain, such as microglia. This process triggers neuroinflammation. In addition, altered intestinal balance can cause signals to be sent directly to the brain via the vagus nerve, thereby inducing the release of neurotransmitters and actively influencing brain activity. For example, a reduction in *Lactococcus* bacteria due to the action of phages can cause a potential reduction in enteric dopamine production, a factor that is relevant in the pathogenesis of PD [16].

Although bacteriophages are predominant, research is also being conducted on eukaryotic viruses that have the capacity to persist in the gut or the CNS, acting directly [17]. Some studies have found alterations in the abundance of eukaryotic viruses, such as Adenovirus or Herpesviridae, in the virome of patients with neurodegenerative diseases. In addition, viral infections known for their neurotropism, such as Epstein–Barr virus or herpes simplex virus (HSV), have been linked to an increased risk of multiple sclerosis (MS) or AD [9,18]. The hypothesis is that the infection, even if controlled, may trigger a chronic and latent immune response that, over time, contributes to neuroinflammation and the accumulation of toxic proteins [19].

This review aims to summarize the latest findings on the crucial role of the microbiota–virus–neurodegeneration axis, examining the primary molecular and cellular mechanisms involved. It also aims to evaluate promising gut-targeted therapeutic strategies for the prevention and management of neurodegenerative diseases.

## 2. The Role of Microbiota in Brain Health

The intestine is considered the largest interface between the body and the external environment. For this reason, it is important to maintain the stability of the intestinal barrier to prevent the entry of luminal substances and pathogens into the internal environment. The composition of the gut microbiome, as well as the activity of intercellular connections, has been demonstrated to be pivotal in determining the stability of the intestinal barrier. The regulation of this process is influenced by hormonal factors, dietary influences, inflammatory mediators, and the enteric nervous system (ENS). The latter, often referred to as the “second brain”, plays a crucial role in regulating intestinal secretion and motility, operating autonomously from the CNS [20].

Several microorganisms inhabit the human intestine, including approximately 10^13^ bacterial cells, collectively known as the “gut microbiota”. The latter is composed of more than 250 species of viruses, fungi, bacteria, and archaea and is considered a dynamic system that continuously evolves. The biological interaction between the host and the gut microbiota is defined as a “close mutualism,” since the latter plays a crucial role in numerous physiological and pathological processes of human life [21,22].

Among the various functions of the microbiota are the synthesis of vitamins, the degradation of dietary polyphenols, the elimination of xenobiotics, and the metabolism of bile acids (BAs) [23,24]. These elements interact bidirectionally with the intestinal microbiota. In fact, the intestinal microbiota produces and regulates these compounds, which in turn influence the composition of the microbiota itself [25].

Moreover, studies in recent years have highlighted the central role of the microbiota in gut–brain communication, giving rise to the microbiota–gut–brain axis, as these metabolic products mediate communication between the two organs—either indirectly through intermediate mediators or directly via the vagus nerve and the ENS [26]. The gut microbiota primarily derives energy from the fermentation of non-digestible carbohydrates, such as fiber, producing SCFAs, including acetate, propionate, and butyrate [27,28]. SCFAs perform several physiological functions, including the regulation of energy metabolism and modulation of the immune response [29].

Furthermore, once in circulation, SCFAs not only serve as energy substrates but also act as signaling molecules within the CNS, crucially influencing processes such as microglial maturation [30,31].

Specifically, circulating SCFAs exert their signaling function primarily by binding to G protein-coupled receptors, notably FFAR2 and FFAR3. These receptors are widely expressed across various cell types, including adipocytes, immune cells, the peripheral nervous system, and the BBB [26].

Furthermore, the gut microbiome actively influences the metabolism of serotonin (5-HT), a key neuromodulator, which together with SCFAs contributes to the modulation of 5-HT synthesis in enterochromaffin cells and potentially influences the expression of the serotonin transporter [32].

In fact, the gut microbiome influences serotonin production by releasing short-chain fatty acids (SCFAs), which modulate its synthesis. The serotonin produced in the gut activates vagal afferent fibers that transmit signals to the nucleus tractus solitarius in the brainstem. The nucleus tractus solitarius receives these signals and projects them to brain regions involved in autonomic regulation, mood, and immune functions. This complex signaling loop underscores the profound regulatory role of gut-derived signals in both brain function and systemic immunity [33].

Neural communication along the axis involves crucial interactions between the microbiota and the Autonomic Nervous System (ANS), the ENS, and the spinal nerves. These interactions are mediated by epithelial cells, enteroendocrine cells, and immune system cells of the intestinal mucosa [34]. At the center of this complex bidirectional communication network lies the vagus nerve (the X cranial nerve). It acts as the main pathway for the transmission of peripheral signals from the intestine to the brain (afferent pathway) and control signals from the brain to the intestine (efferent pathway) [35]. This pathway is essential for autonomic regulation, intestinal motility, and immune responses, as it plays a key role in mediating the effects of gut-derived molecules on the CNS. The vagus nerve is composed of unmyelinated sensory afferent fibers (approximately 80–90% of all nerve fibers) and some myelinated efferent fibers (approximately 10–20%), with the afferent fibers being particularly important in transmitting sensory information from various visceral organs to the brain [33].

Some intestinal vagal afferent fibers act as mechanoreceptors [36], while the remaining vagal afferent fibers act as chemoreceptors and do not come into direct contact with intestinal endoluminal substances. Therefore, they form synapses with the intestinal epithelial cells and the enteroendocrine cells [36]. In this regard, it has been shown that chemoreceptors modulate the relationship between the microbiota and the brain. The injection of microbial colonies, such as *Lactobacillus johnsonii*, into the duodenum leads to the activation of vagal afferent fibers, resulting in improved gastric vagal activity [37].

In addition to the neural (ENS/vagus) and endocrine (metabolites) pathways, the gut–brain axis relies crucially on the immune pathway, represented by the close relationship between the gut microbiota and the Gut-Associated Lymphoid Tissue, as described below.

The bidirectional and symbiotic relationship between the commensal gut microbiota and the host immune system is indispensable for systemic homeostasis. This interaction is dynamic: the immune system constantly modulates the microbiota’s composition, even in the absence of overt inflammation. For instance, a deficiency in crucial components, such as Secretory Immunoglobulin A (sIgA), can lead to the uncontrolled overgrowth of anaerobic bacteria, particularly mucosa-adherent segmented filamentous bacteria (SFBs) belonging to the phylum *Firmicutes* [38]. Metabolites produced by the gut microbiota modulate the metabolism of immune cells through the receptors present on them [39]. These metabolites, produced by various microorganisms, can promote the differentiation and function of immunosuppressive cells. They can also inhibit inflammatory cells, thus contributing to the maintenance of intestinal and systemic immune homeostasis in an individual. The interaction between the immune system and gut microbes is based on mechanisms of recognition, interpretation, and response. Specifically, host recognition occurs through several pathways: SCFAs, tryptophan metabolites, and bile acids. These metabolites play a fundamental role in maintaining intestinal and systemic homeostasis by inhibiting inflammatory immune cells and promoting the differentiation and function of immunosuppressive cells [40]. For example, tryptophan metabolites and bile acids regulate the differentiation and function of regulatory T cells (Tregs), which are critical mediators of immune tolerance and represent a key factor in the effectiveness of immunotherapy and active vaccination [41].

The intestinal microbiota is also involved in the maturation of the innate immune system, as gut bacteria play an important role in this process. In fact, in the absence of microbiota, the functions of neutrophils and dendritic cells are impaired, resulting in a reduced ability to eliminate pathogens and lower secretion of interferon-1 (IFN-1) and interleukin-15 (IL-15). Moreover, the development of myeloid cells in the bone marrow is also affected by the absence of microbiota, which compromises the ability to cope with systemic infections and increases susceptibility to allergies [42]. This highlights how any imbalance in the bidirectional microbiota–immune system relationship can significantly increase the risk of immune-mediated disorders.

Conversely, several studies have demonstrated that certain microbial products act as potent activators of the innate immune system. For instance, lipopolysaccharide (LPS) and its highly potent Lipid A domain, which are abundantly expressed by Gram-negative bacteria, are readily recognized by the innate immune system, leading to a subsequent proinflammatory response [43]. In contrast, other bacteria, such as segmented filamentous bacteria (SFBs), exert distinct, often beneficial, effects on the immune response. SFBs represent a key group of microorganisms that adhere closely to the intestinal epithelium, establishing a mutualistic relationship where they release antigens with a strong immunomodulatory effect on the host [44]. SFBs are therefore associated with a state of immunocompetence. They are linked to the production of IgA by B cells in the gut, as well as in extraintestinal sites, contributing to an effective response against fungal pathogens in the lungs [45,46].

*Akkermansia muciniphila*, which is involved in both innate immunity [47] and the adaptive immune response [48], is one of the other gut microbiota components that is essential to host homeostasis. Depending on personal traits, the makeup of the gut microbiota, and interactions with other environmental factors, this microbe can raise IL-10 levels and alter the fate of T cells and IgG1 levels.

Lastly, some probiotic strains, including *Bifidobacterium lactis* DR10, *Lactobacillus casei Shirota*, and *Lactobacillus rhamnosus* GG, are known to be potent inducers of the immune response by activating a large number of immune cells [49]. Due to these immunomodulatory properties, probiotics have been recommended as potential adjuvants in the management of various pathologies, including cancer [50] and colitis [51] and even in promoting healthy aging [52], thereby supporting their use for the regulation of the immune system.

In the context of neurodegeneration, aging must be critically evaluated as both a driver and a potential confounder. Aging-related immunosenescence and the physiological decline in barrier integrity (inflammaging) create a permissive environment for viral reactivation and systemic translocation. While aging independently contributes to a proinflammatory state, we argue that the gut virome acts as a potent mechanistic driver that exacerbates these age-related vulnerabilities. Specifically, the loss of virome stability in the elderly is not merely a bystander effect of physiological senescence but a catalyst that accelerates the transition to pathological neurodegeneration. By reducing the kinetic barrier for protein aggregation and chronically challenging the BBB, viral elements transform the “physiological” decline of aging into the “pathological” cascade observed in AD and PD [53].

### 2.1. From Dysbiosis to Neuroinflammation: The Role of Barriers and Systemic Circulation

Gut dysbiosis, defined as a loss of equilibrium between beneficial and opportunistic microorganisms, shifts the host–microbiota interaction from a positive state of balance to one that contributes to a chronic inflammatory response [54]. This imbalance is recognized as being at the core of several pathologies, including neurodegenerative diseases, metabolic disorders, and autoimmune conditions. Nevertheless, whether these changes are the cause or consequence remains unclear [55], although recent research seems to indicate that both are correct, as gut dysbiosis contributes to a pathophysiological mechanism [56]. This imbalance is associated with significant gastrointestinal disorders such as Inflammatory Bowel Disease and colorectal cancer. Several Inflammatory Bowel Disease-associated susceptibility genes are correlated with host responses to gut bacteria, highlighting how the gut microbiota also contributes to the pathogenesis of Inflammatory Bowel Disease [57]. In the presence of dysbiosis, bacterial metabolites are significantly altered. Indeed, a study on patients with Inflammatory Bowel Disease showed that the decrease in butyrate-producing bacteria, such as *F. prausnitzii*, led to a low concentration of SCFAs. This reduction affects the differentiation and expansion of Treg cells and the growth of epithelial cells, resulting in the loss of intestinal homeostasis [58]. Compared to healthy individuals, patients with colorectal cancer commonly exhibit lower bacterial diversity and abundance, often with a predominance of Firmicutes and Bacteroidetes. Several studies have shown that dysbiosis, with a consequent increase in bacteria such as *Escherichia coli* (*E. coli*) and ETBF, causes a disruption of the intestinal mucosal barrier. This disruption allows a greater number of bacteria to translocate from the lumen into the tissue. As a result, chronic tissue inflammation occurs, with the release of inflammatory and pro-carcinogenic mediators that increase the risk of developing colorectal cancer [59]. Furthermore, the microbiota constantly communicates with the liver to maintain systemic metabolic balance, a connection known as the Gut–Liver Axis. This communication is regulated by substances such as nutrients, metabolites, and bile acids. When the balance between the microbiota and the liver is disrupted, for example, due to a damaged intestinal barrier, inflammation and liver diseases can develop. For example, in cirrhosis, intestinal microorganisms and their metabolites can worsen inflammation and lead to complications such as hepatic encephalopathy [60].

In recent years, gut microbiota dysbiosis has been revealed to significantly affect brain functions, contributing, often in conjunction with the immune system, to the pathogenesis of neurological conditions, including major depression [61]. The communication along the gut–brain axis is tightly controlled by a series of vascular and epithelial barriers, including the intestinal epithelial barrier, the Gut–Vascular Barrier, the BBB, and the Choroid Plexus Vascular Barrier. The integrity and permeability of these barriers, particularly the BBB and the Plexus Vascular Barrier, are directly influenced by the microbiota. Specific microbial endotoxins of dysbiosis, such as LPSs, can induce systemic inflammation that, in turn, compromises the integrity of the BBB and Plexus Vascular Barrier. The dynamic balance between the gut microbiota, immune cell signaling, and vascular permeability is therefore essential for maintaining the functionality of both intestinal and cerebral barriers. In disorders such as Inflammatory Bowel Disease and Irritable Bowel Syndrome, dysbiosis and increased intestinal permeability (the phenomenon of “leaky gut”) are key features [62]. Damage to the Gut–Vascular Barrier allows microbes, microbial antigens, and inflammatory mediators to translocate into the circulation, initiating low-grade systemic inflammation that can reach distant organs, including the brain. Due to the chronic disruption of mucosal homeostasis and the increase in proinflammatory molecules, Inflammatory Bowel Disease and Irritable Bowel Syndrome are recognized as important comorbidities that significantly increase the risk of developing neurological diseases, including AD, PD, and behavioral disorders [63,64]. Microglia are the brain’s primary immune cells; they help maintain its homeostasis throughout aging. They are considered specialized macrophages of the CNS. Indeed, they act as phagocytes, eliminating pathogens, cellular debris, and Aβ peptides. However, with aging, their ability to phagocytose neurotoxic Aβ plaques diminishes. Consequently, Aβ plaques accumulate and activate microglia, triggering a persistent neuroinflammatory response. It is crucial that microglia receive signals not only from the brain but also from the gut through the vagus nerve. In particular, the afferent fibers of the nerve transmit information to the brain about inflammation and the production of proinflammatory cytokines in the GI tract, thereby also influencing neuroinflammation [65].

**Figure 1:**
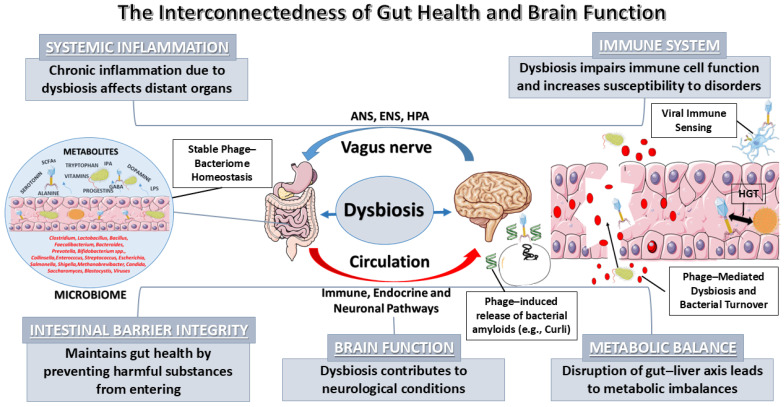
**The interconnectedness of gut health and brain function: communication pathways.** Dysbiosis is the central factor that disrupts the communication pathways along the gut–brain axis. Microbial metabolites (SCFA, GABA, tryptophan) and endotoxins (LPSs) travel via the circulation and the vagus nerve (neuronal pathway) to influence brain function and systemic inflammation. These alterations compromise intestinal barrier integrity and trigger chronic inflammation, leading to neurological conditions and metabolic imbalances (Gut–Liver Axis disruption). The right panel illustrates the mechanisms of neurodegeneration driven by the viral dark matter. Key features include phage-mediated bacterial turnover (lysis of beneficial taxa), HGT of virulence factors, and viral immune sensing. These processes lead to the release of PAMPs and neurotoxic metabolites, which reach the CNS through the vagus nerve and compromised BBB. This image was created using the image bank of Servier Medical Art (available online: http://smart.servier.com/, accessed on 15 December 2025), licensed under a Creative Commons Attribution 4.0 (CC BY 4.0), available online: https://creativecommons.org/licenses/by/4.0/ (accessed on 15 December 2025). BBB: Blood–Brain Barrier; CNS: Central Nervous System; SCFAs: Short-Chain Fatty Acids; IPA: Indolepropionic Acid; GABA: Gamma-Aminobutyric Acid; LPS: Lipopolysaccharide; ANS: Autonomic Nervous System; ENS: Enteric Nervous System; HPA: Hypothalamic–Pituitary–Adrenal Axis; HGT: Horizontal Gene Transfer; PAMPs: Pathogen-Associated Molecular Patterns.

In summary, the equilibrium of the gut microbiota is indispensable for the maturation and ongoing regulation of the immune system, with microbial metabolites acting as fundamental modulators of systemic and central inflammatory responses, as illustrated in detail in Figure 1.

### 2.2. Intestinal Dysbiosis and Neurodegenerative Diseases

Many studies show that the gut–microbiota–brain axis not only affects brain cognition and psychiatric symptoms but is also involved in neurodegenerative diseases such as AD, PD, and MS. The primary question that these studies seek to address is how alterations in the permeability of the BBB and the subsequent development of neuroinflammation and neurodegeneration can be triggered by the microbiota and its metabolites in the context of dysbiosis. This field of enquiry is inherently interdisciplinary, with significant connections to both immunity and chronic systemic inflammation [66]. It is important to emphasize that communication along the microbiota–brain axis occurs throughout the entire lifespan, as observed in neurodevelopmental disorders (e.g., ASD), neurodegeneration (e.g., PD and AD), and behavior (e.g., depression and anxiety). According to recent studies in animals and humans, changes in microbial diversity are associated with negative health outcomes and may lead to alterations in the CNS [67].

#### 2.2.1. The Gut Microbiota in the Pathogenesis of AD: From Imbalance to Amyloid Deposition

AD is a progressive neurodegenerative disorder of the CNS that manifests as a gradual decline in cognitive functions [3]. The core pathological hallmarks of AD are the extracellular deposition of Aβ plaques and the intracellular accumulation of hyperphosphorylated tau protein. The disease is associated with the malfunction of several neurotransmitters, including acetylcholine, dopamine, GABA, serotonin, glutamate, and norepinephrine. The concentration of these neurotransmitters depends on specific bacteria, including *E. coli*, *Bacteroides*, *Eubacterium*, and *Bifidobacterium*. While curative treatments remain elusive, current clinical approaches focus on symptomatic relief, utilizing acetylcholinesterase inhibitors (e.g., donepezil) and NMDA receptor antagonists (e.g., memantine), alongside recent immunotherapies like aducanumab [68].

Several studies have highlighted the presence of dysbiosis in patients with AD. Indeed, an imbalance in the gut microbiota has been observed (an increase in *Bacteroides*, *Escherichia*/*Shigella*, and *Ruminococcus* and a reduction in *Dialister*, *E. rectale*, and *Bifidobacterium*), which influences the progression of AD due to dysregulation in the synthesis of neurotransmitters and their precursors. The resultant low concentration of SCFAs and other neuroactive metabolites contributes to the dysregulation of neurotransmitter synthesis and intestinal–cerebral communication [69,70].

The gut microbiota is implicated in AD pathology via several mechanisms, notably through molecular mimicry. Some bacterial strains can produce functional extracellular amyloid fibers, such as *E. coli*, *Salmonella enterica*, *Bacillus subtilis*, *Mycobacterium tuberculosis*, and *Staphylococcus aureus* [71]. These bacterial strains use these amyloid fibers to form biofilms and adhere strongly to one another to resist destruction by physical and immune factors. The amyloids produced by bacteria differ from CNS amyloids in their primary structure but show similarities in their tertiary structure. This presence of bacterial amyloids in the gut can activate the immune system and may lead to amplified immune responses with the endogenous formation of neuronal amyloids in the brain [72]. An experiment performed on an Amyloid Precursor Protein (APP) transgenic mouse model for AD showed that changes in the composition of intestinal microbial species can promote amyloid deposition. In *APPPS1* mice, a decrease in *Firmicutes* and, at the same time, an increase in *Bacteroides* were observed. Moreover, APP transgenic mice raised under germ-free conditions exhibited a marked reduction in Aβ-associated brain pathology [73]. These findings support the idea that the gut microbiota may activate immune responses capable of initiating amyloid accumulation and thus contributing to the emergence of the characteristic features of AD. In addition to the bacteria residing in the gut, invasive pathogens can also contribute to neurodegenerative processes. Among these, *Mycobacterium leprae* is known for its role in demyelination and nerve fiber damage. This microorganism promotes the onset of infection by altering the microenvironment of Schwann cells and activating apoptotic pathways [74]. *Chlamydia pneumoniae*, typically associated with respiratory tract infections, has also been linked to various CNS disorders, including AD [75]. Liu et al. [76], through the analysis of fecal samples, detected a decrease in microbial diversity in individuals with AD compared to individuals with mild cognitive impairment (MCI) and healthy individuals. In particular, in AD patients, they observed a significant decrease in bacteria of the phylum Firmicutes, while an increase in the phylum Proteobacteria, especially bacteria of the family Enterobacteriaceae, was noted compared to fecal samples from healthy individuals. Their analysis also revealed a significant increase in Bacteroidetes during the preclinical stage of AD compared to the group of healthy individuals. Similarly, Sheng et al. indicated that, in the early stages of AD, there is intestinal dysbiosis characterized by an increased number of Bacteroidetes [77].

Hao et al. conducted studies on germ-free mice or mice treated with antibiotics to deplete the gut microbiota and demonstrated that these mice exhibited reduced Aβ deposition and decreased neuroinflammation, highlighting the crucial role of the microbiota in AD [78]. Conversely, interventions targeting the microbiota show promise: a clinical study demonstrated that a 12-week probiotic treatment in AD patients led to improved cognitive function and a reduction in the serum levels of proinflammatory cytokines, highlighting the potential benefits of modulating the gut microbiota to reduce neuroinflammation and slow AD progression [79].

#### 2.2.2. PD: From Dysbiosis to α-Syn Aggregation and Neurodegeneration

PD is the second most common neurodegenerative disorder, characterized primarily by the dysfunction and loss of dopaminergic neurons. The pathological hallmark of PD is the abnormal aggregation of the insoluble α-syn protein into Lewy bodies within vulnerable neuronal and glial populations of the CNS. The high frequency and early occurrence of gastrointestinal symptoms, such as constipation, prolonged intestinal transit time, and small intestinal bacterial overgrowth, together with the detection of α-syn aggregates in the ENS and the reduced lifetime risk of Parkinson’s disease following full truncal vagotomy, support the hypothesis that the pathophysiology of Parkinson’s disease may originate in the gut [80].

Patients with PD consistently exhibit distinct gut dysbiosis patterns compared to healthy individuals. Key findings across various studies include a significant deficiency in SCFA-producing bacteria, which possess anti-inflammatory properties. Notably, there is a marked decrease in taxa from *Roseburia*, *Faecalibacterium*, the *Lachnospiraceae* ND3007 group, *Prevotellaceae*, *Blautia*, *Coprococcus*, and *Lachnospira*, which are major butyrate producers. Conversely, an increased abundance is frequently observed in potentially proinflammatory taxa, such as *Akkermansia*, *Catabacter*, *Lactobacillus*, *Bifidobacterium*, *Bifidobacteriaceae*, *Ruminococcaceae*, *Verrucomicrobiaceae*, and *Christensenellaceae* [81]. These alterations in microbial composition, particularly the reduced abundance of anti-inflammatory SCFA producers (such as taxa from the *Lachnospiraceae* family and *Faecalibacterium prausnitzii*), are associated with a worse clinical profile, including motor and cognitive deficits, and are already present in patients with early-onset PD who have never received treatment. Furthermore, dysbiosis extends beyond bacteria, showing a reduction in total viral richness but an increase in *Lactobacillaceae* abundance [82]. Recent studies on the gut microbiome in PD patients have shown no differences in fungal abundance but often exhibit a reduction in total viral richness and a reduction in bacteria that produce SCFAs, primarily butyrate [83].

Notably, within the gut microbiota, bacteria such as *Enterococcus* and *Bacillus* are known for their ability to synthesize dopamine. Microbial alterations also affect the effectiveness of drug treatment. In fact, the intraduodenal infusion of Levodopa–Carbidopa intestinal gel is associated with an increase in the relative abundance of the *Enterobacteriaceae* family. This increase is problematic, as it has been observed that PD patients infected with *Helicobacter pylori* show reduced absorption of L-dopa [84]. A study conducted on transgenic rats that overexpressed α-syn revealed that intestinal dysbiosis progressed with aging. Crucially, short-term antibiotic treatment was able to reduce the expression of α-syn in the forebrain [85]. These findings support the hypothesis of a causal role for the gut microbiota in the progression of the disease.

Intestinal bacteria are capable of producing an extracellular amyloid that, through the mechanism of molecular mimicry, can trigger the aggregation of α-syn [86]. For example, *E. coli* can produce curli, an extracellular amyloid protein with structural and biophysical properties similar to human pathological amyloids. It can also activate the innate immune system and facilitate α-syn aggregation and neuroinflammation [87]. Noteworthily, bacterial functional amyloids, such as curli fibers, are well-known triggers for host protein misfolding; however, the virome acts as a critical upstream modulator of this process. Through phage-mediated bacterial turnover, bacteriophages induce the lytic destruction of commensal bacteria, leading to the massive and synchronized release of intracellular bacterial amyloids and DNA into the intestinal lumen. This process significantly increases the bioavailability of amyloidogenic seeds that can then undergo translocation across the compromised gut barrier, modulating amyloidogenic processes in this way [88]. This mechanism is supported by clinical evidence in PD, where shifts in the gut phageome have been linked to the depletion of beneficial bacteria and the potential release of proinflammatory and amyloidogenic components [89]. The role of dysbiosis in the pathogenesis of PD is also linked to the impairment of the intestinal barrier. Hirayama et al. [90] performed a study on intestinal permeability and inflammation caused by gut dysbiosis associated with PD. Their research found an increase in *Akkermansia* and a decrease in SCFA-producing bacteria, which may facilitate the exposure of the intestinal neural plexus to toxins such as LPSs and pesticides. This can lead to abnormal α-syn fibril aggregation and the development of Lewy bodies. Moreover, gut microbes could help predict who might be at risk of developing PD; for example, a reduced abundance of SCFA-producing microbes could indicate the likelihood of a future transition to PD in patients with idiopathic rapid eye movement sleep behavior disorder [91]. Alterations in the microbiota, such as an increased abundance of *Christensenellaceae* or *Oscillospira*, are associated with a higher risk of developing PD. This suggests that specific changes in the microbiome could potentially be used to diagnose the disease at an early stage. This diagnostic potential is reinforced by evidence that dysbiosis is already present in patients with early-onset PD, even before receiving any drug treatment [92]. Analyses carried out on large cohorts of Italian and international patients have outlined a consistent pattern of dysbiosis in PD. A marked reduction in the abundance of the *Lachnospiraceae* family (known for its production of SCFA) has been found. One study reported that lower levels of *Lachnospiraceae* and higher levels of *Christensenellaceae* and *Lactobacillaceae* were associated with a worse clinical profile, characterized by motor and cognitive deficits [82]. This evidence highlights the need for further studies on the microbiome to monitor disease progression and identify which changes in microbial composition play a causal role or influence disease status; indeed, alterations in *Enterobacteriaceae* and *Lachnospiraceae* were associated with greater disease severity and motor deficits. Functional analysis revealed changes in the metabolic pathways of SCFAs and amino acids, as well as pathways promoting intestinal inflammation [93].

Consistent with the role of microbiota in this disease, clinical trials using fecal microbiota transplantation from healthy donors to patients with PD have shown that it is possible to reduce motor symptoms and improve non-motor symptoms, such as sleep quality and quality of life, and alleviate anxiety, depression, and constipation in patients with PD [94]. This evidence highlights the need for further studies on the microbiome to monitor disease progression and identify which changes in microbial composition play a causal role or influence disease status.

#### 2.2.3. Gut Dysbiosis and the Gut–Brain Axis in MS Pathogenesis

MS is a chronic, inflammatory, and neurodegenerative disease caused by autoimmune reactions that progressively demyelinate the CNS and the spinal cord. It appears to arise when autoreactive T cells cross the BBB and trigger specific cascades within the CNS, leading to inflammation and axonal degeneration, although it remains unclear what causes T-cell activation [95].

The risk factors are highly complex and arise from the interaction between genetic variants and environmental factors. Numerous lines of evidence show that the gut microbiota of patients with MS is altered: several studies, in fact, have detected differences in the intestinal microbiome profile compared to healthy individuals [96]. This appears to promote the onset of a proinflammatory state. Moreover, dysbiosis increases the permeability of the BBB. This process leads to the inflammation of the CNS and neurodegeneration, which may ultimately contribute to the development of MS [97].

Several clinical studies have examined the gut microbiota composition of patients with MS and found that, compared with healthy controls, these patients showed increased concentrations of *Pedobacteria*, *Flavobacterium*, *Pseudomonas*, *Mycoplana*, *Acinetobacter*, *Eggerthella*, *Dorea*, *Blautia*, *Streptococcus*, and *Akkermansia*. At the same time, the amounts of *Prevotella*, *Bacteroides*, *Parabacteroides*, *Haemophilus*, *Sutterela*, *Adlercreutzia*, *Croprobacillus*, *Lactobacillus*, *Clostridium*, *Anaerostipes*, and *Faecalibacterium* were reduced in their gut microbiota, indicating that dysbiosis is indeed present in patients with MS. The authors suggest that treating dysbiosis appears to reduce inflammation and reactivate the immune system by modulating the effect of T lymphocytes in humans [98]. The scientific literature suggests that intestinal bifidobacteria may play a protective role in the onset of neuroinflammatory diseases, including MS and Guillain–Barré syndrome. The inflammatory role of T helper cells is central to these diseases: the cytokines IFN-gamma and IL-17, produced by Th1 and Th17 cells respectively, are known to contribute to the development of both conditions. Confirming this, a clinical study has shown that the levels of circulating Th1, Th17, and Th22 cells, together with the plasma levels of IL-17 and IL-22, are significantly elevated in both patients with Guillain–Barré syndrome and those with relapsing–remitting MS in acute relapse [99]. Cekanaviciute et al. identified certain bacteria associated with MS and showed that they can influence T-cell-mediated adaptive immune responses, creating a proinflammatory environment both in vitro and in vivo. Their findings suggest that targeting the microbiota could represent a potential therapeutic strategy for MS. The study of the microbiome in 71 patients with relapsing–remitting MS (RR-MS) and 71 healthy subjects showed that MS patients had a higher abundance of *Akkermansia muciniphila* and *Acinetobacter calcoaceticus*, while *Parabacteroides distasonis* was less abundant. These findings not only confirm the association between microbiota and MS but also suggest that targeting the microbiota (i.e., its therapeutic manipulation) could represent a potential treatment strategy for MS [100].

Among the environmental factors influencing MS, microbes, and the substances they secrete or the toxins they produce, play a significant role in its pathophysiology [101]. Furthermore, the gut microbiome of patients with active MS displays a composition that differs from that of individuals in remission, whose microbial composition more closely resembles that of healthy individuals [102]. In preclinical models, germ-free mice develop an attenuated MS-like disease, whereas mice receiving the intestinal microbiota from MS patients exhibit more severe experimental autoimmune encephalomyelitis and display a lower proportion of anti-inflammatory regulatory T cells compared to mice treated with the microbiome of healthy individuals. It was striking to observe that transplanting the intestinal microbiota from MS twins into germ-free animals, genetically susceptible to developing experimental autoimmune encephalomyelitis, was sufficient to induce the disease in vivo, with a significantly higher incidence than that seen when transplanting the microbiota from healthy twins. Interestingly, the immune cells of mice that received samples from MS patients produced lower amounts of IL-10 compared to those of animals colonized with the microbiota from healthy twins. This suggests that a deficient regulatory response is key, as the neutralization of IL-10 in mice inoculated with healthy fecal samples also led to an increased incidence of experimental autoimmune encephalomyelitis [103].

This important finding highlights how the human microbiome can induce specific changes in the immune system, potentially contributing to or resulting from the onset of MS. However, it remains unclear whether this role is crucial for the initiation and progression of the disease. In this context, the differences in the microbiota of MS patients compared to healthy controls have attracted considerable interest.

Collectively, these findings confirm that gut dysbiosis is not merely an association but a functional driver of neuroinflammation and neurodegeneration, strongly supporting the microbiota as a prime therapeutic target for these complex CNS disorders. The principal findings from clinical and preclinical studies investigating the role of the gut microbiota in the pathogenesis of AD, PD and MS are summarized in Table 1.

## 3. Viral Infections and Neurodegeneration

Neurodegenerative diseases (NDDs), including AD, PD, amyotrophic lateral sclerosis (ALS), and MS, are characterized by progressive neuronal loss, pathological protein aggregation, and cognitive or motor decline, representing a major global public health challenge [104]. Their etiology is multifactorial, with aging and genetic predisposition being the most significant risk factors. However, many viral infections can contribute to the onset or acceleration of neurodegenerative processes.

Pathologically, NDDs share the common feature of accumulated and aggregated cellular proteins. For instance, PD is characterized by the formation of Lewy bodies composed of aggregated α-syn, while AD is defined by two signature hallmarks: extracellular Aβ plaques and intracellular neurofibrillary tangles formed by hyperphosphorylated tau protein [105].

Recent evidence suggests that bacteriophages can cross the intestinal epithelial barrier through transcytosis and reach the systemic circulation and lymphatic system. From there, they may act as direct immunomodulators, interacting with pattern recognition receptors (PRRs) even in distal sites like the CNS [9]. Viral infections have been demonstrated to exacerbate the degeneration of the brain through the disruption of metabolic and immune homeostasis. In particular, they have the capacity to initiate a series of neurodegenerative processes, encompassing chronic neuroinflammation, oxidative stress, mitochondrial dysfunction and impaired proteostasis. In addition to these factors, the process is further exacerbated by an augmentation in protein aggregation. The collective effect of these phenomena is the development of neurodegenerative pathology [106]. The viruses that have been identified as playing a significant role in such processes are HSV type 1 (HSV-1), Epstein–Barr virus (EBV), Human immunodeficiency virus type 1 (HIV-1), and SARS-CoV-2 [107].

### 3.1. Specific Neurotropic Viruses and Pathological Links to NDDs

Neurotropic viruses have the ability to infect cells of the nervous system, remaining in a latent state. These viruses can enter the CNS through hematogenous spread, axonal transport, or through the olfactory epithelium [108]. Once inside the CNS, they can infect neurons, astrocytes, microglia, or endothelial cells, causing neuroinflammation, oxidative stress, and the disruption of neuronal homeostasis. Viral infections can profoundly disrupt protein homeostasis and increase the susceptibility of cells to protein misfolding [106].

The neurotropic viruses studied include members of various families of RNA viruses, as well as some DNA viruses and retroviruses. Some DNA viruses are from the Herpesviridae family. HSV-1/HSV-2 and varicella-zoster virus (VZV) establish lifelong latency in peripheral neurons, with a particular predilection for the trigeminal and olfactory ganglia [109,110]. Under stressors like immunosuppression or psychological stress, the virus can reactivate and move back to the periphery, causing cold sores or genital lesions. Alternatively, it can infiltrate the CNS, leading to herpes simplex encephalitis, a condition particularly associated with HSV-1. Even without classic reactivation, HSV-1 can cause encephalitis in vulnerable individuals such as newborns or those who are immunocompromised [111]. During latency, HSV-1 maintains its genome as an episome within the nucleus of neurons. The primary function of the latency-associated transcript is to maintain the inactive state of the virus and to protect infected neurons from undergoing cell death. In cases where HSV-1 causes encephalitis, the infection results in viral replication within neurons and glial cells. This results in necrosis, apoptosis, and significant inflammation, particularly in the temporal lobes and limbic regions. The BBB becomes disrupted partly due to the overproduction of matrix metalloproteinases-9 by activated microglia [112]. This worsens damage to the barrier, allows immune cells to enter the brain, and contributes to swelling and tissue necrosis. Proinflammatory cytokines like IL-6 and TNF-α further increase brain swelling and injury. Even after the acute phase, survivors may experience long-term issues such as cognitive problems and seizures. These issues arise from ongoing inflammation and neuron loss. HSV-2 tends to cause meningitis more often than encephalitis under stress or when the immune system is weak [113]. Latent HSV-1 DNA and proteins have been identified in the hippocampus and temporal cortex of patients with AD [114]. HSV-1 infection promotes Aβ deposition and tau hyperphosphorylation, key hallmarks of AD pathology, likely mediated through the activation of host kinases and oxidative stress pathways [115]. VZV can reside latently in the brain and, in the case of reactivation, cause indirect damage [116]. VZV infection in HSV-1 latent neural cells leads to HSV-1 reactivation, inducing an increase in Aβ and p-tau. In fact, epidemiological studies suggest the potential involvement of VZV in AD/dementia [117].

EBV, another member of the Herpesviridae family, primarily infects B lymphocytes and epithelial cells but can indirectly affect the CNS through immune-mediated mechanisms. Structural similarities between the viral capsid protein EBNA1 and CNS autoantigens, including myelin basic protein, may underlie this effect. EBV has been strongly associated with MS: although EBV does not directly infect neurons, its systemic immune effects contribute to neuroinflammation and demyelination [118]. Recent evidence indicates that CD4^+^ T cells show a preferentially heightened response to EBNA1, characterized by an expansion of EBNA1-specific central memory Th1 precursors and Th1 effector memory cells, without a parallel involvement of Th17 cells. In individuals with MS, EBNA1-specific T cells more often cross-react with myelin antigens than with autoantigens not associated with the disease, secrete IFN-γ, and exhibit a polyfunctional profile typical of chronic viral infections. Taken together, these findings support the idea that EBNA1-specific CD4^+^ T cells, via the cross-recognition of myelin antigens, may actively participate in the pathogenesis of MS [119]. The Retroviridae family also includes neurotropic viruses such as HIV-1 and human T-lymphotropic virus type I (HTLV-I). HIV-1 can cross the BBB early during infection. HIV-1-infected cells release viral proteins such as gp120 and Tat, which induce excitotoxicity, oxidative stress, and apoptosis in adjacent neurons. Tat activates MMPs, enzymes that degrade extracellular matrix components and weaken the BBB. Gp120-mediated interactions with CXCR4 and CCR5 receptors on endothelial cells further contribute to BBB disruption [120]. Chronic microglial activation and the release of proinflammatory cytokines contribute to HIV-associated neurocognitive disorders, characterized by cognitive decline and neuronal loss. HTLV-1 is the causative agent of HTLV-1-associated myelopathy/tropical spastic paraparesis, a chronic inflammatory disorder of the spinal cord. In contrast to other infections, HTLV-1 does not directly damage the CNS; rather, it accomplishes this by infecting CD4^+^ T cells. The aforementioned cells infiltrate the CNS, thereby instigating a potent inflammatory response through the secretion of proinflammatory cytokines, such as TNF-alpha and IFN-gamma. This inflammatory process damages the BBB, allowing for the further infiltration of immune cells, which leads to chronic inflammation and damage to motor neurons in the spinal cord. In addition, the viral protein Tax stimulates MMP production through inflammatory pathways, promoting the breakdown of the extracellular matrix and disruption of tight junctions, facilitating the paracellular entry of infected cells [121,122].

Among RNA viruses, those belonging to the *Flaviviridae* family are known to cause encephalitis or meningitis and use different strategies to cross the BBB. Patients with West Nile Virus, Japanese Encephalitis Virus, and Dengue Virus infections show a significant risk of developing Parkinsonian symptoms. The expression of α-syn was found to be increased in primary neurons and in the brains of patients with acute West Nile Virus encephalitis [123,124]. In contrast, Japanese Encephalitis Virus infection damages areas rich in dopaminergic neurons, leading to a marked decline in catecholamines (such as dopamine and norepinephrine), which is related to impaired locomotor activity [125]. Like other neurotropic viruses, Dengue induces pathology in the CNS through the induction of cellular stress and neuroinflammation [126]. CNS manifestations associated with Dengue infection include fever, encephalitis, and meningitis [127]. Dengue has also been linked to potential long-term neurodegenerative effects. Clinical and experimental studies indicate that patients with Dengue infections are at significant risk of developing Parkinsonian symptoms [128].

Other neurotropic RNA viruses belong to the Coronaviridae family, such as severe acute respiratory syndrome coronavirus 2 (SARS-CoV-2), the causative agent of COVID-19. Although SARS-CoV-2 primarily affects the respiratory system, it can invade the CNS through the olfactory pathway or through infected endothelial and immune cells. The virus binds to ACE2 receptors expressed on neurons and glia, causing endothelial alteration, microglial activation, and BBB disruption. Post-mortem and neuroimaging studies have revealed cortical atrophy, white matter damage, and increased neuroinflammation markers [129]. Notably, the spike and nucleocapsid proteins of SARS-CoV-2 have been associated with the acceleration of α-syn overexpression and aggregation, which is related to the formation of Lewy bodies in vitro [130,131].

Collectively, these neurotropic viruses, summarized in Table 2, share several convergent mechanisms of neurotoxicity, including the induction of chronic inflammation, mitochondrial impairment, and perturbation of protein homeostasis. Their ability to persist or reactivate within neural tissues highlights viral infection as a potential initiating or amplifying factor in the pathogenesis of neurodegenerative diseases.

### 3.2. Pathogenic Mechanisms of Neurotropic Viruses: From Neuroinvasion to Chronic Neurodegeneration

Neurotropic viruses have evolved various strategies to penetrate the CNS, overcoming the highly selective barriers that protect the brain and spinal cord. Hematogenous spread and crossing the BBB comprise the most common route, in which the virus circulates in the blood (viremia) and crosses the endothelium of the cerebral vessels. This passage can occur via a paracellular route, in which the virus crosses the space between endothelial cells due to the breakdown of tight junctions, often mediated by systemic inflammation that releases proinflammatory cytokines such as TNF-α and IL-6. The virus can use a transcellular pathway, directly infecting the endothelial cells of the BBB or crossing them via a process of transcytosis (vesicular transport). Another common mechanism is the so-called “Trojan horse” mechanism. The virus enters the CNS by hiding inside infected immune cells (such as monocytes, macrophages, or T lymphocytes) that naturally migrate across the barrier for immune surveillance. Examples that use these modes of entry are HIV-1, HTLV-1, West Nile Virus, Japanese Encephalitis Virus, Dengue Virus, SARS-CoV-2, VZV, and EBV (via infiltration of infected B cells) [106,132]. Some viruses can completely bypass the bloodstream and the BBB by infecting peripheral nerve endings (sensory or motor) or neuromuscular junctions, using the cell’s transport machinery to travel backward along axons to the cell bodies of neurons in the spinal cord or brain [133]. This entry mechanism is called retrograde axonal transport and is often used by viruses such as HSV-1, HSV-2, VZV, West Nile Virus, and Japanese Encephalitis Virus [108]. The olfactory route, on the other hand, allows for rapid and direct access to the brain by bypassing the BBB. Viruses infect the olfactory sensory neurons located in the nasal cavity; from there, the virus travels along the olfactory nerve to the olfactory bulb and then spreads to deeper brain structures. This route of entry can be used by viruses such as HSV-1, HSV-2, VZV, West Nile Virus, Japanese Encephalitis Virus, and potentially SARS-CoV-2 [108,134].

Within this framework of neuroinvasion, Alphaviruses (e.g., Chikungunya virus and Equine Encephalitis viruses) represent a significant paradigm of viral neurotropism. These viruses exploit both hematogenous and olfactory routes to access the CNS, where they interact with specific host cell receptors to initiate infection. For instance, Chikungunya virus primarily utilizes the Mxra8 receptor for entry [135], while Venezuelan Equine Encephalitis Virus targets LDLRAD3 [136] or heparan sulfate on neuronal surfaces.

Once the virus has gained entry, its subsequent spread within the CNS is not merely stochastic; it is guided by the axonal transport machinery. This process involves the hijacking of dynein and kinesin motors for retrograde and anterograde movement, respectively, allowing the virus to navigate interconnected neural circuits. This active spread is a critical step in establishing the chronic neuroinflammatory state that characterizes neurodegeneration [137]. The primary receptors and specific neuroinvasive mechanisms for key neurotropic viruses, including Alphaviruses, are summarized in Table 3.

#### 3.2.1. Innate Immune Recognition and Neuroinflammation

The transition from an acute viral infection to a chronic neurodegenerative process involves a complex interplay between viral persistence and the host’s immune and metabolic responses. Below, the primary mechanisms driving this progression are discussed.

Inflammation is a fundamental defense mechanism of the innate immune system, activated in response to tissue damage or infection. Its primary role is to eliminate pathogens and promote tissue repair. Innate immune cells, including monocytes, macrophages, mast cells, dendritic cells, and microglia in the CNS, detect danger signals originating from both pathogens and injured host cells. These signals include PAMPs, which are characteristic of bacteria, viruses, and other pathogens, and damage-associated molecular patterns (DAMPs), which are released by host cells. PAMPs and DAMPs are recognized by PRRs, such as toll-like receptors (TLRs), NOD-like receptors (NLRs), and other intracellular sensors. TLR3 is highly expressed in the Purkinje neurons of the cerebellum in human brains affected by viral encephalitis, ALS, or AD [138].

The activation of pattern recognition receptors stimulates immune cells to produce reactive oxygen species (ROS) and reactive nitrogen species (RNS), which contribute to the elimination of pathogens. This activation also triggers the release of proinflammatory cytokines (IL-1β, TNF-α, IL-6), chemokines, and growth factors while enhancing the phagocytosis of cellular and tissue debris. Under normal conditions, the brain is safeguarded by physical and immunological barriers, including the BBB, which restricts pathogen entry. The CNS tightly regulates immune activity to protect neurons, which are irreplaceable once lost. Excessive inflammation can severely damage non-renewable cells, such as neurons and oligodendrocytes. For this reason, adaptive immune responses in the CNS are generally limited [139]. Nevertheless, some microorganisms are capable of bypassing these protective mechanisms.

When viruses reach the CNS, microglia become activated and release inflammatory signals to help contain and control the infection. Neuroinflammation involves not only the activation of microglia and astrocytes but also the recruitment of peripheral immune cells, such as inflammatory monocytes, macrophages, and antigen-specific T lymphocytes, that can enter the CNS when the BBB becomes more permeable. However, if this inflammatory response becomes excessive, it can lead to collateral damage to surrounding neural cells [140].

#### 3.2.2. Mitochondrial Impairment and Oxidative Stress

The brain depends on a constant and considerable energy supply to maintain proper function, and even small metabolic disruptions can interfere with normal neuronal activity. In AD, one of the earliest and most consistent alterations involves impaired cerebral energy metabolism [141]. Under physiological conditions, glucose is the primary energy substrate for the adult human brain. Neuronal energy production relies mainly on mitochondria, which generate ATP through oxidative phosphorylation. During this process, a small proportion of electrons leaks from the electron transport chain, leading to the formation of ROS. At low concentrations, ROS function as important signaling molecules, but excessive ROS production results in oxidative stress. Although mitochondria possess antioxidant defense systems, they remain highly vulnerable to ROS-mediated damage. Once impaired, mitochondria produce less ATP and generate even more ROS, establishing a self-perpetuating cycle. As a result, oxidative stress can act both as a cause and a consequence of mitochondrial dysfunction [142].

#### 3.2.3. Dysregulation of Autophagy and Proteostasis

In NDDs, autophagy is often impaired. Under normal conditions, autophagy enables neurons to eliminate damaged proteins and organelles, thereby preserving cellular homeostasis. In disorders such as AD, PD, and ALS, this process becomes progressively less efficient [143]. As a result, misfolded or aggregated proteins accumulate, dysfunctional mitochondria are not adequately removed, and autophagic vesicles may build up due to defective fusion with lysosomes. The gradual decline in autophagy ultimately contributes to neuronal dysfunction and cell death. Viruses can also disrupt autophagy to support their own replication and survival. Some viruses inhibit the fusion of autophagosomes with lysosomes, preventing their degradation and instead exploiting these vesicles as replication platforms [144].

#### 3.2.4. Viral Latency and Reactivation

Neurotropic viruses can establish latent infections within neurons, where the viral genome persists but remains transcriptionally silent. During latency, most viral genes are suppressed through structural modifications of viral DNA and interactions with host chromatin-regulating proteins. Viral and host-derived factors, including non-coding RNAs and microRNAs, contribute to maintaining this quiescent state by inhibiting viral gene expression and supporting neuronal survival. Although latency can persist for long periods, various stressors or physiological triggers can disrupt this balance, enabling the virus to reactivate and resume replication [145]. Some viruses produce proteins or peptides that closely resemble those of the host. This phenomenon, known as molecular mimicry, allows viruses to evade immune detection by appearing similar to host structures. However, this similarity can mislead the immune system, causing it to target the host’s own cells that share these antigenic features, ultimately leading to autoimmunity. This mechanism is especially relevant in neurodegenerative and neuroinflammatory diseases. Neurotropic viruses such as EBV can express epitopes that mimic neuronal or glial proteins, eliciting immune responses that damage myelin or other components of the CNS. Such cross-reactive responses may initiate or exacerbate autoimmune processes that contribute to disorders like MS and other autoimmune encephalopathies [146]. CD4^+^ T cells are capable of recognizing and responding to both EBV-derived peptides and self-antigens, including myelin basic protein (MBP), anoctamin 2, alpha-crystallin B, and glial cell adhesion molecules [147]. MBP, a self-protein present in the myelin sheath, can trigger autoreactive T cells in MS patients when presented by antigen-presenting cells that also display EBV-derived peptides. Compared with healthy individuals, MS patients exhibit higher frequencies of CD4^+^ T cells that are cross-reactive with both EBV peptides and MBP [148].

**Figure 2:**
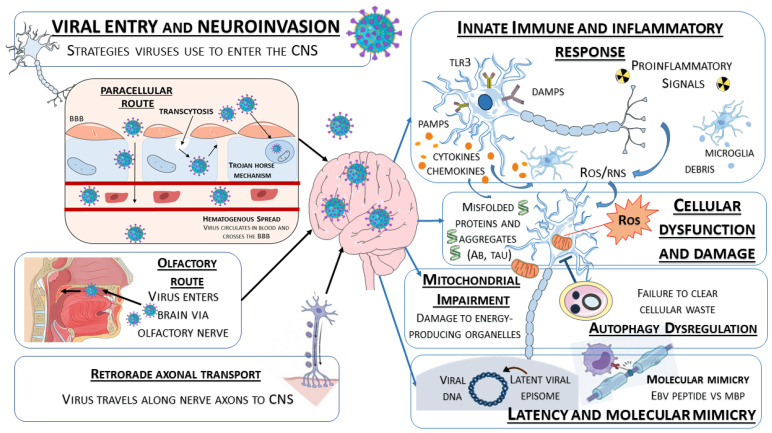
**Pathogenetic mechanisms of neurotropic viruses: from neuroinvasion to chronic neurodegeneration.** This figure summarizes the fundamental stages through which viral infection leads to neuronal damage. Neuroinvasion: Viruses can penetrate the CNS by crossing the BBB via the paracellular route (tight junction breakdown), transcytosis, or the “Trojan horse” mechanism within infected immune cells. Other entry routes include retrograde axonal transport from neuromuscular junctions and the direct olfactory pathway. Innate Immune and Inflammatory Response: Once inside the CNS, viral PAMPs and DAMPs released by damaged cells are recognized by PRRs, such as TLR3, on neurons and microglia. This triggers microglial activation and the release of proinflammatory cytokines (TNF-alpha, IL-6), chemokines, and ROS/RNS. Cellular Dysfunction and Damage: Chronic inflammation and oxidative stress lead to mitochondrial dysfunction (driving further ROS production), impaired autophagy, and the subsequent accumulation of misfolded proteins and pathological aggregates, such as Aβ and tau. Latency and Molecular Mimicry: Certain viruses establish latent infections (viral episome in the nucleus). Furthermore, molecular mimicry between viral peptides (e.g., EBV) and self-antigens (e.g., MBP in myelin) can elicit autoimmune responses. This image was created using the image bank of Servier Medical Art (available online: http://smart.servier.com/, accessed on 15 December 2025), licensed under a Creative Commons Attribution 4.0 (CC BY 4.0), available online: https://creativecommons.org/licenses/by/4.0/ (accessed on 15 December 2025). Aβ: Amyloid-beta; BBB: Blood–brain barrier; CNS: Central nervous system; DAMPs: Damage-associated molecular patterns; EBV: Epstein–Barr virus; IL-6: Interleukin-6; MBP: Myelin basic protein; PAMPs: Pathogen-associated molecular patterns; PRRs: Pattern recognition receptors; RNS: Reactive nitrogen species; ROS: Reactive oxygen species; TLR3: Toll-like receptor 3. ⊣: inhibition/block.

Noteworthy, the transition from an acute viral infection to a chronic neurodegenerative process involves a complex interplay between viral persistence and host immune responses. As summarized in Figure 2, neurotropic viruses employ multiple strategies to penetrate the CNS, triggering a pathogenetic cascade that includes neuroinflammatory activation, mitochondrial dysfunction, altered proteostasis, and autoimmune phenomena, which collectively lead to irreversible neuronal damage.

### 3.3. The Gut Virome: A Novel Player in the Gut–Brain Axis and Neurodegeneration

Many of the bacterial shifts documented in neurodegenerative diseases remain partially unexplained because research has often overlooked the “viral dark matter” of the gut. This vast, uncharacterized portion of the microbiome, primarily composed of bacteriophages, acts as a hidden regulator of microbial ecology, suggesting that what we perceive as primary bacterial dysbiosis is often a secondary effect of viral predation and genomic manipulation [149]. Prevailing research has historically focused on the direct neuroinvasion of these pathogens. However, recent studies have revealed that the gut virome, as the core of this dark matter, exerts a substantial indirect influence on brain health. Eukaryotic viruses and, primarily, bacteriophages that infect intestinal bacteria make up the human gut virome. In recent years, there has been an increased focus on the significant role of the gut virome in human health and disease. The majority of gut-associated bacteriophages are members of the order *Crassvirales* (formerly *Caudovirales*), which is very prevalent in the intestinal environment and mainly infects Bacteroidetes bacteria [150]. The gut virome in healthy people varies greatly from person to person but stays largely constant over time [13]. However, there is mounting evidence that the composition of the virome can be changed by lifestyle factors and is altered in a number of disease conditions. Gut viruses are important regulators of the microbial ecosystem, in addition to their function in regulating bacterial abundance. The virome indirectly affects the synthesis of microbial metabolites, such as SCFAs, which are critical for preserving the integrity of the gut barrier and promoting BBB function, by influencing the dynamics of bacterial populations. Furthermore, immune signaling pathways that support neuroimmune balance can be modulated by intestinal viruses through direct interaction with host immune cells. If this viral balance is upset, it could lead to systemic inflammation and have detrimental effects on the central nervous system. The disruption of this viral equilibrium may promote systemic inflammation and negatively affect CNS function, highlighting the virome as an important component of the gut–brain axis [151].

Zuo et al. examined 98 COVID-19 patients with varying degrees of disease severity and contrasted them with 78 non-COVID-19 controls in order to evaluate changes in the gut virome over time during SARS-CoV-2 infection. In fecal samples, COVID-19 patients showed an enrichment in environmentally derived eukaryotic DNA viruses and a decrease in several bacteriophage groups compared to uninfected individuals. Interestingly, up to 30 days after clinical recovery, these virome changes remained in the gut. Individuals with COVID-19 generally had a virome that was enriched in environmental and eukaryotic viruses, while non-COVID-19 controls had a higher abundance of bacteriophages. These compositional variations may be linked to SARS-CoV-2 infection and probably represent alterations in the host immune system and gut microbiota brought on by infection [152].

Because bacteriophages control bacterial populations through predator–prey interactions, the gut virome plays a crucial role in determining microbial diversity. This helps to maintain a balanced ecosystem that includes bacteria, viruses, and the host. Rather than looking at bacterial abundance in isolation, the ratio between specific bacteriophages and their bacterial hosts—the viral-to-bacterial ratio—emerges as a more precise biomarker for neurodegenerative progression. A dysregulated ratio can lead to a chronic state of “leaky gut”, facilitating the translocation of neurotoxic metabolites and viral particles into the bloodstream [9]. The stability and resilience of the gut microbiome, which is becoming more widely acknowledged as a significant modulator of neurophysiological processes and general systemic health, are supported by this dynamic equilibrium. Therefore, maintaining both physical and mental health depends on maintaining a well-controlled intestinal microbiome, which is fueled by coordinated interactions among the virome, the bacterial community, and the host immune system [153].

Recent clinical studies have shown that patients with AD have significant alterations in the microbial composition and gut-derived metabolites. AD patients have lower levels of key microbiological metabolites, such as SCFAs, particularly butyrate, and neuroprotective tryptophan derivatives. These changes may promote amyloid aggregation and impair synaptic plasticity. Concurrently, there have been reports of increased intestinal permeability and bile acid levels, which have been connected to systemic inflammation and neuronal damage. Reduced microbial diversity in AD patients has been found through microbiome profiling, which is typified by an increase in proinflammatory *Bacteroidetes* and a decrease in beneficial taxa within *Firmicutes* (such as *Ruminococcaceae*) and *Actinobacteria* (such as *Bifidobacterium*). The phage-mediated depletion of beneficial taxa, such as *Faecalibacterium prausnitzii*, should not be viewed solely as a bacterial shift; it is likely driven by a “kill-the-winner” dynamic, where specific bacteriophages bloom in response to high bacterial density, leading to massive lysis and the subsequent loss of anti-inflammatory metabolites like butyrate [154,155]. Furthermore, the expansion of pathobionts (potentially pathological microorganisms that coexist as harmless commensals under normal conditions) in AD and PD might be facilitated by prophage induction. Stress conditions within the gastrointestinal tract of patients with neurodegenerative diseases can trigger the transition of lysogenic phages to the lytic cycle, which not only alters the bacterial community structure but also releases PAMPs that exacerbate neuroinflammation [156]. In addition to direct lysis, the virome influences neurodegeneration through horizontal gene transfer (HGT). Bacteriophages can act as vehicles for virulence factors and metabolic genes, effectively “reprogramming” host bacteria. In the context of AD and PD, this viral-mediated HGT may enhance the fitness of pathobionts or increase the production of neurotoxic bacterial metabolites, further fueling chronic neuroinflammation [157]. Significant remodeling of the gut virome with decreased viral diversity has also been found in amyloid-positive AD patients, particularly in the *Siphoviridae* family and the *Uroviricota* phylum. Because such phages control *Lactococcus* populations and affect the ratio of L-lactic acid, which supports neural metabolism and memory, to D-lactic acid, which may worsen neurodegenerative processes, the selective loss of *Lactococcus*-infecting phages is particularly interesting. Moreover, a higher abundance of *Caudoviricetes* phages has been associated with better executive function, suggesting that virome alterations represent an important, yet underexplored, contributor to gut–brain axis dysfunction and cognitive decline in AD [158]. Similarly, gut virome dysbiosis with increased viral richness and diversity is a hallmark of PD. Bacteriophage families like *Siphoviridae* and *Myoviridae*, which specifically target bacterial genera like *Fecalibacterium* and *Alistipes*, are enriched in PD patients. This phage-mediated depletion of butyrate-producing bacteria leads to a failure in intestinal epithelial integrity, triggering the translocation of viral particles and bacterial toxins (such as PAMPs) into the systemic circulation. In PD, this shift could accelerate the aggregation of α-syn in the enteric nervous system through the inflammatory environment created by massive viral lysis. This mechanism supports the “bottom-up” hypothesis, where neuroinflammation and protein misfolding spread from the gut to the brain via the vagus nerve or across a compromised BBB [9]. Conversely, the virome of healthy individuals is typically dominated by phages linked to *Bacteroides* and *Prevotella*, which maintain a stable microbial ecosystem and support neuroprotective functions. The loss of helpful bacteria that produce short-chain fatty acids may worsen the function of the gut barrier and increase inflammation. Together, these results point to a potential role for altered viral–bacterial interactions in neurodegenerative diseases and imply that the gut virome is an important but little-studied factor in the dysfunction of the gut–brain axis [19].

## 4. The Viral Modulation of the Gut Microbiota and Neuroimmune Homeostasis

The interaction between viruses and the host extends beyond direct pathogenicity, leading to a significant reorganization of the commensal microbial community, a phenomenon known as “viral dysbiosis”. Following SARS-CoV-2 infection, significant changes in the diversity and composition of the intestinal microbiota have been consistently documented. These changes appear early in the course of the disease and become the most pronounced between the second and third weeks [159]. This viral signature is characterized by a reduction in butyrate-producing families, such as *Ruminococcaceae* and *Lachnospiraceae*, as well as a decrease in members of the phylum *Firmicutes*, particularly the genus *Faecalibacterium*, which correlates with systemic inflammatory responses and disease severity [160]. Conversely, an increase in opportunistic bacterial species has frequently been observed [161].

In addition to the gut microbiota, SARS-CoV-2 infection is associated with significant changes in the oral microbiota. Ma et al. demonstrated that alterations detected in oropharyngeal swabs correlate with inflammatory responses and are associated with disease severity. Moreover, comparative analyses of patients with influenza and COVID-19 have revealed distinct oral and intestinal microbiota profiles, highlighting disease-specific microbial signatures [162].

Evidence from animal models suggests that the antiviral activity against hepatitis B virus (HBV) may be mediated, at least in part, by immune modulation driven by the intestinal microbiota [163]. Recent clinical studies have further supported an association between gut microbiota composition and chronic HBV infection, as well as HBV-related cirrhosis and hepatocellular carcinoma [164]. In patients with chronic HBV infection without liver injury, a reduction in *Bacteroides* has been reported. In contrast, HBV-related liver diseases are characterized by progressive and complex alterations in the intestinal microbiota, although the reported patterns of dysbiosis vary among studies. In the same way, systemic infections such as HBV and HCV show how viruses can alter the gut ecology. These intricate changes show a change in microbial output that may have an impact on the homeostasis of distal organs, such as the CNS. Studies investigating the intestinal microbiota in hepatitis C virus (HCV)-infected patients have predominantly reported a reduction in microbial diversity [165], although some reports have described increased diversity, indicating heterogeneity in microbiota alterations associated with HCV infection.

These examples underscore the shift from a “bacteria-centric” view toward a holistic understanding of the gut–brain axis, where viral agents act as key players in neurobiology.

### 4.1. Microbiota–Immune System Crosstalk in Antiviral Defense

The gut microbiota is a key regulator of antiviral immunity, mediating constant crosstalk with the host immune system. This interaction is not merely a local gastrointestinal event but a critical component of the neuroimmune axis, where microbial signals calibrate the activation threshold of CNS-resident immune cells, such as microglia. Commensal microbes support the development and functional readiness of innate and adaptive immune cells, including dendritic cells, macrophages, and T lymphocytes.

Microbial-derived signals, such as metabolites and molecular patterns, help prime interferon responses and modulate the activity of pattern recognition receptors. In this way, the microbiota enhances effective antiviral defenses while preventing excessive inflammatory reactions that could damage host tissues. Antimicrobial peptides produced by bacteria, also known as bacteriocins, are synthesized by many bacterial species and have long been considered one of the key characteristics of probiotics. In addition to their antimicrobial activity, some bacteriocins appear to be able to act against viruses, although these mechanisms of action are still not fully understood [166]. In some cases, bacteriocins act before the virus enters human cells, blocking viral entry. One example is duramycin, produced by *Streptomycetes*, which can prevent Zika virus from entering cells by blocking its coreceptor TIM1 [167]. In other cases, bacteriocins do not stop viral entry but instead limit viral damage and reduce the number of new viral particles produced by interfering with the late stages of the viral replication cycle. For example, a bacteriocin produced by *Lactobacillus delbrueckii* does not affect the early stages of influenza virus infection but reduces the production of viral proteins in infected cells [168]. Similarly, subtilosin, produced by bacteria of the genus Bacillus, interferes with the late stages of infection by HSV-1 [169] and HSV-2 [170]. These microbiota-derived antiviral peptides may act as a biochemical barrier, preventing the neuroinvasion of systemic pathogens, given the growing evidence connecting latent viral infections, like HSV, to the pathophysiology of AD and cognitive decline.

Early exposure to antibiotics has been shown to impair antiviral immunity. Research in gnotobiotic mice, germ-free animals treated with antibiotics, demonstrates that the disruption of commensal bacteria delays the development of lymphoid tissues, disturbs immune cell homeostasis, and increases susceptibility to gastrointestinal infections and inflammatory diseases [171]. In a murine model, maternal antibiotic administration during pregnancy and lactation induced significant alterations in the gut microbiota of both mothers and offspring, leading to higher and faster mortality in pups following viral infection [172]. Moreover, antibiotic-treated mice display compromised innate and adaptive antiviral responses, with markedly delayed viral clearance after exposure to systemic lymphocytic choriomeningitis virus or mucosal influenza virus. This effect is attributed to the essential role of commensal bacteria in providing signals that calibrate the activation threshold and sensitivity of the innate antiviral immune system [171]. Consequently, antibiotic-induced dysbiosis during critical life stages could act as a catalyst for inflammaging, where compromised immune priming leads to persistent low-grade inflammation and the reduced clearance of neurotropic threats in the aging brain.

Studies in animals and in vitro models have also highlighted interactions between noroviruses and the intestinal microbiota [173]. In vitro experiments indicate that human noroviruses can infect B cells more effectively in the presence of bacteria expressing histo-blood group antigens, suggesting that bacteria associated with the intestinal epithelium may facilitate viral entry. Correspondingly, the antibiotic-mediated depletion of the microbiota in mice prevents persistent norovirus infection [174].

Similar interactions between viruses and the microbiota have been reported for poliovirus, reovirus, murine mammary tumor virus, and rotavirus [175]. LPSs derived from intestinal bacteria have been shown to enhance the stability and infectivity of poliovirus particles, suggesting that certain commensal bacteria can facilitate enteroviral infections [176]. These contrasting roles, where the microbiota can either thwart or facilitate viral entry, underscore the complexity of the viral–bacterial–host triad, highlighting the need for a holistic approach to understand how individual microbial signatures influence brain health.

Conversely, specific members of the microbiota may confer protection against viral infections. In immunocompromised mice, segmented filamentous bacteria were found to protect against rotavirus infection independently of immune cell involvement [177]. Additionally, in vitro studies suggest that the microbiota can reduce norovirus attachment to host cells [178]. Viral infections such as norovirus or rotavirus are also associated with decreased microbial diversity, and clinical observations in Ghanaian patients with diarrhea indicate significant alterations in microbiota composition following infection [179].

Another important finding is that certain microorganisms of the resident microbiota play a protective role against viral infections of the upper respiratory tract [180]. Recent studies have shown that susceptibility to influenza A (H3N2) and B infections is linked to the composition of the nasal and pharyngeal microbiota. Interferon induction, associated with innate immunity against the genus Staphylococcus in the nasal cavity, is considered the molecular mechanism underlying the protective function of the microbiota against viruses [181]. Understanding these molecular mechanisms is vital for developing personalized interventions that leverage the protective capacity of the virome–microbiota axis to preserve cognitive function.

### 4.2. The Concept of Viral Dysbiosis: The Impact on Microbiota Homeostasis and Barrier Integrity

Viral dysbiosis represents a pathological shift where viral-induced alterations in the microbiota compromise the structural integrity of biological barriers, creating a permissive environment for systemic inflammation to reach the CNS. Numerous lines of evidence demonstrate that variation in microbiome structure and function impacts viral immunity. It has been observed that certain bacterial species, such as *Lacticaseibacillus casei* and *Bifidobacterium adolescentis*, indirectly contribute to the preservation of gastrointestinal barrier integrity by producing metabolites associated with the reduced expression of the rotavirus toxin NSP4, which during infection disrupts the structure and function of tight junctions [182]. The preservation of these tight junctions is a critical first line of defense; their disruption not only facilitates enteric symptoms but may also initiate the systemic circulation of proinflammatory cytokines that eventually challenge the BBB’s integrity.

Acute viral gastroenteritis is an enteric viral infection that causes diarrhea. This infection often leads to bacterial dysbiosis, meaning an imbalance in the composition of the gastrointestinal tract microbiota. The processes that drive this dysbiosis and the consequences following viral infection are not yet fully understood.

Some changes in the microbiota are closely linked to the viral pathogen. For example, a decrease in *Bifidobacterium* is more commonly observed in infections caused by rotavirus and human norovirus, while it is less pronounced in infections caused by human Astrovirus. Rotavirus infection causes a marked loss of bacterial diversity, partly due to an increase in *Gammaproteobacteria* [183]. This marked loss of bacterial diversity and the expansion of pathobionts like *Gammaproteobacteria* are indicative of a “fragile” microbiome state, which has been increasingly associated with the low-grade systemic inflammation observed in age-related cognitive decline [184].

Enteric viral infections can also affect the microbiota in other parts of the body. In children with symptomatic HFMD (hand, foot, and mouth disease), an increase in *Streptococcus* spp. has been observed in the oral microbiome, positively correlated with viral RNA levels in saliva. One study also reported alterations in the salivary virome, including both human viruses and phages, compared to healthy subjects [185].

Another example of viral infection outside the gastrointestinal tract is respiratory syncytial virus (RSV), which can modify the intestinal microbiota. The link between respiratory viral infection and intestinal dysbiosis has been demonstrated in murine influenza models and is mediated by adaptive immune cells [186].

These findings confirm that viral-induced dysbiosis is a systemic phenomenon; by altering the intestinal landscape, even respiratory or oral viruses can indirectly influence the gut–brain axis, potentially exacerbating neuroinflammatory pathways through the recruitment of distal adaptive immune cells.

### 4.3. Functional Evidence of the Virome–Microbiota–CNS Interaction

An increasing amount of preclinical and clinical data supports the shift from merely seeing correlations to comprehending the functional dynamics of the virome–microbiota–brain axis. Preclinical research employing germ-free or antibiotic-treated murine models has been crucial in showing how the integrity of the BBB and the maturation of microglia are compromised by the lack or depletion of commensal signals. “Viral dysbiosis” serves as the main stressor in these models [187]. For example, systemic viral challenges in mice with altered microbiota cause an exaggerated neuroinflammatory response, which is typified by the recruitment of peripheral immune cells into the CNS and a failure to eliminate neurotropic threats [188].

Beyond indirect bacterial modulation, viral elements uniquely contribute to amyloidogenesis through molecular structural scaffolding. Viral capsids and nucleic acids released during viral replication can act as heterologous templates for the cross-seeding of host proteins. These viral particles provide a highly ordered surface that reduces the kinetic barrier for the nucleation of α-syn and Aβ, thereby accelerating their aggregation. Furthermore, neurotropic viruses can directly impair the host’s proteostatic machinery, such as the autophagy–lysosome pathway, further hindering the clearance of these amyloid aggregates in the CNS [88]. Complementing these mechanistic insights, the high-throughput sequencing of patient cohorts has significantly expanded clinical evidence. Amyloid-positive AD patients have a unique viral signature that sets them apart from healthy controls, according to a seminal metagenomic study [158]. These results imply that changes in the enteric virome act as “silent intruders” that cause systemic inflammation and may occur before clinical symptoms. Similarly to this, research on PD and ALS has shown a strong correlation between the presence of viral components that alter the host’s neuroimmune response and gut dysbiosis.

A recent study of patient cohorts has provided a more granular understanding of these viral signatures. In AD, Aβ^+^ individuals exhibit a significant ~9% reduction in the dominant bacteriophage phylum Uroviricota, characterized by a decrease in Caudoviricetes/Siphoviridae and a simultaneous 11% increase in *Poxviridae.* Notably, higher Siphoviridae abundance has been positively associated with better cognitive performance, particularly executive function, while specific *Lactococcus* phages (e.g., bIL285, bIL286, and P335) appear significantly depleted in AD patients. In contrast, PD patients show a distinct pattern of increased viral richness and diversity. This includes an enrichment in Myoviridae, Siphoviridae, Podoviridae, and p-crAss-like phages, alongside a depletion of Quimbyviridae. These PD-associated viral operational taxonomic units (vOTUs) primarily target key commensal bacteria such as *Alistipes*, *Faecalibacterium*, and *Oscillibacter*, whereas in healthy controls, vOTUs are mainly linked to *Prevotella* and *Bacteroides*. This suggests that the viral “dark matter” actively drives the loss of beneficial taxa in PD through targeted predation [9]. Similarly, in MS, recent viromic analyses have identified significant dysbiosis characterized by a shifted virome–bacteriome ratio. Specifically, a depletion in the diversity of the *Caudoviricetes* class has been linked to a reduction in butyrate-producing bacteria. Moreover, recent evidence demonstrated an enrichment in crAss-like phages in RR-MS patients, which negatively correlates with the abundance of beneficial taxa such as *Bacteroides* and *Prevotella*. This viral-driven microbial shift facilitates the translocation of PAMPs and potentially viral antigens, exacerbating systemic inflammation and the activation of peripheral immune cells that contribute to CNS demyelination [189].

Multi-omic integration is becoming more and more necessary for the validation of this triadic link [15]. Researchers have started mapping the production of neuroactive metabolites (like SCFAs and neurotransmitter precursors) influenced by bacteriophage–host interactions by combining transcriptomics and metabolomics with metagenomics, which identifies the viral and bacterial populations. These cutting-edge technologies make it possible to identify “pathogen-associated signaling biomarkers,” which offer a path forward for early diagnosis and the creation of tailored therapeutic interventions. The modulation of the gut virome is a promising frontier for disease-modifying therapies in neurodegeneration, as it is becoming possible to establish causal links through the monitoring of longitudinal cohorts [190].

**Figure 3:**
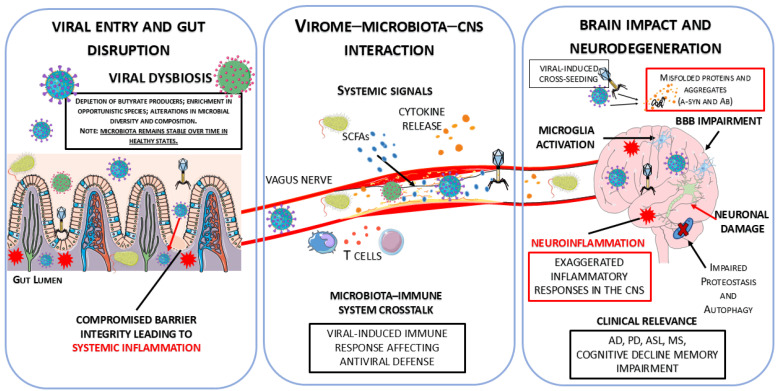
**The virome–microbiota–brain axis in neuroimmune homeostasis.** This figure illustrates the systemic impact of viral infections on the gut–brain axis, highlighting the progression from local dysbiosis to neuroinflammation. Gut Lumen—Viral Dysbiosis (**Left** Panel): Exposure to viral pathogens (e.g., SARS-CoV-2, norovirus, HBV) triggers “viral dysbiosis,” characterized by a significant reduction in beneficial commensal bacteria, such as butyrate-producing *Faecalibacterium*, and an overgrowth of opportunistic pathobionts. This shift disrupts the local biochemical environment, including the production of antimicrobial bacteriocins and essential metabolites. Intestinal Barrier—Systemic Transmission (**Center** Panel): Viral-induced alterations lead to compromised barrier integrity (leaky gut). The breakdown of tight junctions facilitates the translocation of proinflammatory cytokines and microbial-derived signals into the systemic circulation and along the vagus nerve, transforming a localized enteric event into a systemic inflammatory response. CNS—Neuroinflammation (**Right** Panel): Chronic systemic inflammation challenges the BBB and promotes the activation of CNS-resident immune cells, particularly microglia. Viral particles further contribute via structural scaffolding, accelerating the cross-seeding and aggregation of α-syn and Aβ; simultaneously, neurotropic viruses impair the autophagy–lysosome pathway, hindering the clearance of these neurotoxic aggregates. This persistent neuroinflammatory state, often referred to as “inflammaging,” drives neuronal damage and contributes to the pathophysiology of neurodegenerative disorders, including AD, PD, ALS and MS. This image was created using the image bank of Servier Medical Art (available online: http://smart.servier.com/, accessed on 15 December 2025), licensed under a Creative Commons Attribution 4.0 (CC BY 4.0), available online: https://creativecommons.org/licenses/by/4.0/ (accessed on 15 December 2025). AD: Alzheimer’s Disease; Aβ: β-Amyloid; α-syn: Alpha-Synuclein; ALS: Amyotrophic Lateral Sclerosis; BBB: Blood–Brain Barrier; CNS: Central Nervous System; HBV: Hepatitis B Virus; PD: Parkinson’s Disease; SCFA: Short-Chain Fatty Acid; MS: Multiple Sclerosis.

In summary, the complex interplay between viral-induced dysbiosis, the breakdown of biological barriers, and the subsequent activation of neuroimmune pathways highlights the gut virome as a strategic driver of brain health and neurodegeneration (Figure 3).

## 5. Future Prospects and Therapeutic Implications

In conclusion, while bacterial changes are the most visible sign of dysbiosis in AD and PD, the gut virome acts as the “silent intruder” that orchestrates these shifts, making it a primary driver of brain aging. This review highlights how the gut virome is becoming a key player in the gut–brain axis, changing the paradigm in neurobiology. The recent literature is shifting the “bacteria-centric” view towards a holistic understanding that includes the viral realm, opening up unexplored avenues for the early diagnosis and treatment of NDDs. Future prospects and therapeutic implications in the fight against neurotropic viruses are focusing on understanding the microbiota–virus–brain axis, as it has emerged that commensal bacteria can enhance microglial activity and protect the CNS from viral replication. Since the virome varies from individual to individual and can be influenced by lifestyle, the use of multi-omic approaches (integrating metabolomics, transcriptomics and proteomics) is essential. In fact, sequencing phage populations in the gut has revealed new dynamics in viral populations, capable of regulating the overall composition of the microbiome [151]. Therefore, the multi-omic approach will allow us to accurately map how specific bacteriophages influence the production of neuroactive metabolites, identifying “viral signatures” that can serve as predictive biomarkers for AD or PD years before the onset of clinical symptoms [19,158]. In this regard, multi-omic approaches have already successfully modeled the pathological effects of viruses such as Zika, suggesting that these technologies can identify new molecular targets for personalized treatments. At the same time, personalized viromics based on the sequencing of patient samples is considered necessary to identify adaptive viral mutations and host genetic polymorphisms that influence susceptibility to neuroinvasion. In this field, one of the challenges is the inability to cultivate and isolate many of these phages in vitro, a problem that is being addressed bioinformatically using metagenomic techniques and advanced sequencing methods. Bioinformatic programs are adopting new strategies and increasing functionality, speed and effectiveness to identify as many phages as possible. Several new phages, including crAss-like phages, gubaphages and Lak phages, have been identified through metagenomic data mining. The gut virome is a complex feature that is essential for maintaining human health [153].

New therapeutic strategies involve the use of precision probiotics or new-generation “psychobiotics” [191]. These aim to restore deficient bacterial taxa and also act as viral balance stabilizers, counteracting the expansion of pathogenic phages (e.g., those affecting *Faecalibacterium*) or promoting the growth of bacteria that produce L-lactic acid and butyrate [192]. The integration of antiviral components into these formulations could prevent alterations in the microbiota induced by systemic infections such as SARS-CoV-2. In addition to the development of precision probiotics, another innovative frontier concerns the development of synthetic microbial communities (SynComs), designed not only to rebalance the intestinal flora but also to act as therapeutic vehicles capable of releasing specific molecules, such as neurotransmitter precursors (serotonin, GABA) or immunomodulatory molecules, thereby reducing chronic neuroinflammation [193].

In this context, fecal microbiota transplantation emerges as a powerful tool for restoring microbial diversity and the integrity of biological barriers (intestinal and blood–brain). Preliminary studies have shown potential in reducing neuroinflammation in animal models of AD and PD, reducing Aβ accumulations and improving motor and cognitive functions [194,195,196,197]. Notably, new therapeutic strategies include Fecal Virome Transplantation, which involves the selective transfer of the viral fraction (mainly phages) from healthy donors [198]. This approach may offer greater safety than full fecal microbiota transplantation, reducing the risks of bacterial pathogen transfer and allowing for an ecological reshuffling of the gut aimed at supporting brain homeostasis [190].

**Figure 4:**
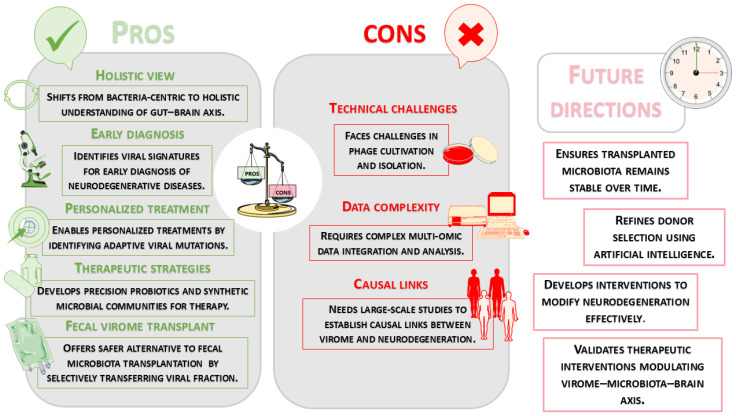
**A summary of the pros, cons, and future directions of virome-based research and therapy for neurodegenerative diseases.** This infographic provides a balanced overview of the therapeutic landscape for the virome–microbiota–brain axis in the context of neurodegeneration. Pros (**Left** Panel): This section highlights the shift toward a more holistic understanding of the gut–brain axis, moving beyond a purely bacteria-centric view to include the role of the virome. It emphasizes the potential for early diagnosis through the identification of viral signatures and the development of personalized treatments by analyzing adaptive viral mutations. Additionally, it underscores the potential of therapeutic strategies such as precision probiotics and Fecal Virome Transplant, which offers a safer, more selective alternative to traditional fecal microbiota transplantation by focusing on the viral fraction. Cons (**Center** Panel): This section outlines the significant technical and analytical barriers to clinical translation. Technical challenges are paramount, specifically the difficulties associated with phage cultivation and isolation. Safety concerns are also highlighted, including the risk of unintentional “viral dark matter” transfer and the horizontal gene transfer of antibiotic resistance genes during Fecal Transplant. The immense data complexity also presents a hurdle, as it requires the integration and analysis of complex multi-omic datasets. Furthermore, a lack of longitudinal data limits our understanding of long-term neuroimmune modulation and the stability of the transplanted virome. Finally, a critical gap remains in establishing clear causal links between the virome and neurodegeneration, a task that necessitates large-scale studies to move beyond simple correlation. Future Directions (**Right** Panel): This section outlines forward-looking strategies for the field. Key prospects include ensuring the long-term stability of transplanted microbiota and leveraging artificial intelligence to refine donor selection processes. Another promising avenue is the development of targeted interventions designed to modify the course of neurodegeneration effectively. The ultimate goal is the validation of therapeutic interventions that modulate the virome–microbiota–brain axis, establishing a robust and scalable model for treating complex neurological disorders. This image was created using the image bank of Servier Medical Art (available online: http://smart.servier.com/, accessed on 15 December 2025), licensed under a Creative Commons Attribution 4.0 (CC BY 4.0), available online: https://creativecommons.org/licenses/by/4.0/ (accessed on 15 December 2025).

Despite the promising potential of probiotics and fecal microbiota transplantation, significant limitations and safety concerns must be addressed to ensure clinical viability. A primary risk involves the unintentional transfer of the donor’s virome during fecal microbiota transplantation. This “viral dark matter” (comprising not only bacteriophages but also potentially opportunistic eukaryotic viruses) can persist in the recipient’s gut, triggering maladaptive long-term immune responses or altering the host’s neuroimmune tone [199]. These risks are critically amplified in elderly patients, where age-related inflammaging and the physiological decline in intestinal barrier integrity facilitate the systemic translocation of proinflammatory viral antigens and opportunistic pathogens [53]. Crucially, bacteriophages can act as vectors for the horizontal gene transfer of antibiotic resistance genes and virulence factors, potentially creating reservoirs of resistant pathobionts within the recipient [200]. Furthermore, as highlighted in our analysis of therapeutic “cons” (Figure 4), technical hurdles such as the difficulty in phage cultivation and isolation hinder the development of standardized microbial cocktails, leading to the current reliance on raw fecal material with poor quality control. Clinical outcomes also remain inconsistent due to the “donor effect,” where the specific viromic and metabolomic profile of a donor may not align with the recipient’s gut environment. This mismatch can result in the translocation of proinflammatory viral antigens across a compromised intestinal barrier, potentially exacerbating systemic inflammation [199]. Consequently, to confirm whether virome alterations are a cause or consequence of neurodegeneration, it is imperative to initiate large-scale integrated longitudinal studies. Such studies are essential for understanding the long-term stability of transplanted microbiota and for refining donor selection protocols through artificial intelligence, ensuring safe and effective interventions that can effectively modify the course of neurodegeneration. By monitoring cohorts of patients from the prodromal stages to disease progression, it will be possible to establish causal links and validate the effectiveness of therapeutic interventions based on the modulation of the virome–microbiota–brain axis [190].

The overall therapeutic landscape, including the current advantages, technical challenges, and future milestones of this field, is summarized in Figure 4.

## Figures and Tables

**Table 1:** **The gut–brain axis and dysbiosis in major neurodegenerative and neuroinflammatory diseases.** This table summarizes the key scientific evidence linking alterations in the gut microbiota (dysbiosis) to the pathogenesis of major CNS diseases such as AD, PD, and MS. This table analyzes the role of the microbiota through the gut–brain axis, highlighting causal mechanisms and clinical consequences.

Type of Study	Model/Species	Experimental Design	Mechanism of Action	Observed Effect (Behavior/Physiology)	Disease Stage	Refs.
Key Studies on Gut Microbiota and AD						
Observational (Human)	AD Patients/Healthy Controls	Analysis of fecal samples and serum/plasma biomarkers.	Dysbiosis (↑ proinflammatory bacteria, ↓ SCFA producers) leading to systemic inflammation.	Significant differences in microbiota composition (e.g., ↑ Bacteroides, Escherichia/Shigella; ↓ Dialister, Bifidobacterium).	Varies (Preclinical, MCI, Established AD)	[69,70]
Experimental (Animal)	APP Transgenic Mice (AD Model)	Raising under germ-free conditions or antibiotic treatment.	Absence/depletion of microbiota, leading to a reduction in pro-amyloidogenic signals.	Marked reduction in Aβ deposition and neuroinflammation in the brain.	Early Pathogenesis	[73]
AD Patients/In Vitro Models	-	Detection of invasive pathogens (e.g., *C. pneumoniae*, *M. leprae*).	Pathogen-induced invasion/inflammation contributing to neuronal damage (e.g., demyelination, neuroinflammation).	Detection of specific pathogens associated with CNS disorders and AD.	Varies (Risk Factor/Progression)	[74,75]
Observational (Human)	Individuals with AD or MCI and Healthy Controls	Fecal sequencing analysis (microbiota diversity).	Specific dysbiosis: Alterations in microbial diversity (↓ biodiversity, ↑ proinflammatory taxa) influencing the gut–brain axis communication.	Decreased microbial diversity in AD individuals compared to MCI and healthy controls. ↓ Firmicutes and ↑ Proteobacteria (Enterobacteriaceae); ↑ Bacteroidetes in the preclinical stage.	Varies (Preclinical, MCI, Established AD)	[76]
Observational (Human)—Prediction of Cognitive Decline by Neuroimaging Techniques and the Application in Diagnosis and Treatment of Preclinical AD; ClinicalTrials.gov: NCT03370744	Sino Longitudinal Study on Cognitive Decline SILCODE) (Preclinical AD, MCI, Healthy Controls)	Cross-sectional analysis; gut microbiota sequencing combined with plasma Aβ measurement.	Specific dysbiosis in early-stage AD. The combined microbiota and plasma Aβ profile serves as a potential diagnostic index for early AD identification.	The combined index (microbiota profile + plasma Aβ) showed high capability to identify preclinical AD. A significant increase in Bacteroidetes was noted in the early AD stages.	Preclinical and Early Stage	[77]
Experimental (Animal)	APP Transgenic Mice (AD Model)	Microbiota depletion via antibiotics; study of the role of IL-17a.	Microbiota depletion reduces peripheral inflammatory signals, leading to a reduction in Th17 cells and the cytokine IL-17a. This mitigates neuroinflammation and Aβ pathology in the brain.	↓ Aβ deposition and ↓ neuroinflammation in the brain of antibiotic-treated mice. The effect is partly mediated by the reduction in IL-17a levels.	Early and Progressive Pathogenesis	[78]
Intervention (Human)	AD Patients	Probiotic administration (e.g., 12 weeks).	Microbiota modulation and reduction in the serum levels of proinflammatory cytokines.	Improved cognitive function and reduced systemic inflammation.	Established AD	[79]
Key Studies on Gut Microbiota and PD						
Observational (Human)	AD Patients/Healthy Controls	Fungal sequencing analysis (mycobiome).	Specific dysbiosis within the fungal kingdom and its association with inflammation and PD pathology.	Differences in mycobiome composition: found a significant association between specific fungal taxa and PD pathology.	Varies	[83]
Experimental (Animal)	Transgenic Rats Overexpressing Human α-syn	Observation over aging; comparison with short-term antibiotic treatment.	α-syn overexpression drives gut dysbiosis and alters microbial metabolites over time, directly impacting CNS pathology.	Progressive gut dysbiosis observed with aging. Short-term antibiotic treatment reduced α-syn expression in the forebrain.	Pathogenesis	[85]
Mechanistic (Molecular/Microbial)	Host Neurodegeneration Models	Genome-wide screen; identification and functional validation of bacterial component.	Curli (bacterial amyloid fibril) promotes host neurodegeneration by structurally facilitating the aggregation of endogenous proteins (α-syn).	Identified a specific bacterial amyloid component (curli) that activates the innate immune system and drives pathological protein aggregation in the host.	-	[86]
Mechanistic (Molecular/Animal)	Aged Fischer 344 Rats; *C. elegans*	Experimental exposure to the curli protein produced by *E. coli*.	Curli (bacterial amyloid) acts as a seed or template to cross-seed and accelerate the misfolding and aggregation of host α-syn.	Exposure to Curli enhanced α-syn aggregation and inflammation in the brain; curli activated the innate immune system.	-	[87]
Observational (Human)	PD Patients, iRBD Patients, Healthy Controls	Fecal sequencing analysis; comparison of SCFA-producing bacteria.	SCFA deficiency contributes to PD pathology but may not be the primary trigger in the very early, prodromal phase (iRBD).	↓ SCFA-producing microbiota found in established PD patients but not in iRBD patients. This suggests that SCFA deficiency is more closely linked to manifest PD.	Prodromal (iRBD) and Established PD	[91]
Observational (Human)	PD Patients/Healthy Controls (Colonic Mucosal Biopsies and Fecal Samples)	Comparison of mucosal and fecal microbiota compositions using sequencing. Predictive metagenomics performed.	Proinflammatory dysbiosis triggers inflammation-induced α-syn misfolding and PD pathology. High levels of genes for LPS biosynthesis and Type III secretion systems.	Fecal and mucosal microbiota are significantly different. ↓ anti-inflammatory, butyrate-producing bacteria (*Blautia*, *Coprococcus*, *Roseburia* in feces; *Faecalibacterium* in mucosa). ↑ proinflammatory *Proteobacteria* (*Ralstonia* in mucosa).	Established PD	[92]
Observational (Human)	Idiopathic PD, Progressive Supranuclear Palsy, Multiple System Atrophy, Healthy Controls (Fecal Samples)	16S rRNA gene sequencing (N = 350); stratified by disease duration and adjusted for confounders (e.g., diet).	The microbiota acts as an environmental modulator of PD pathogenesis and contributes to interindividual variability in clinical features.	↓ *Lachnospiraceae* (the only significant difference). The association of ↓ *Lachnospiraceae* and ↑ *Lactobacillaceae*/*Christensenellaceae* correlates with worse clinical profile (higher cognitive impairment, gait disturbances, and postural instability).	Established PD, Atypical Parkinsonism	[82]
Observational (Human)	PD Patients (N = 80)/Healthy Controls (N = 72) from Central Italy (Fecal Samples)	16S rRNA gene sequencing; predictive metagenomic analysis; correlation with clinical scores.	Proinflammatory environment and reduced biosynthesis of amino acids (precursors of physiological transmitters).	↓ *Lachnospiraceae*. ↑ *Lactobacillaceae*, *Enterobacteriaceae*, *Enterococcaceae*. ↓ *Lachnospiraceae* and ↑ *Enterobacteriaceae* correlated with increased disease severity and motor impairment. Functional analysis showed variations in pathways for SCFA/amino acid metabolism and LPS biosynthesis.	Established PD	[93]
Intervention (Human)	PD Patients	Preliminary clinical trial (fecal microbiota transplantation from healthy donors).	Modulation of the gut microbiota to restore eubiosis and reduce inflammatory signals and α-syn synthesis.	Improvement in motor symptoms and non-motor symptoms (e.g., constipation, quality of life).	Varies/Established	[94]
Key Studies on Gut Microbiota and MS						
Observational/Intervention (Human)	Guillain–Barré Syndrome Patients, RR-MS Patients in Acute Relapse and Healthy Controls	Comparison of circulating T cells (Th1, Th17, Th22) and plasma cytokines (IL-17, IL-22); observation during IVIg treatment in Guillain–Barré syndrome patients.	Elevated levels of Th cell subsets (Th1, Th17, Th22) are central to the inflammatory response in Guillain–Barré syndrome and MS relapse.	Elevated levels of circulating Th1, Th17, and Th22 cells compared to HC. Plasma IL-17 and IL-22 were markedly elevated.	Acute Relapse MS and Guillain–Barré Syndrome	[99]
Observational (Human/Animal)	RR-MS Patients (N = 71)/Healthy Controls (N = 71); Mouse Models with Experimental Autoimmune Encephalomyelitis	16S rRNA gene sequencing in humans; in vitro assays with human T cells; fecal microbiota transplant to mouse models with experimental autoimmune encephalomyelitis to test disease exacerbation.	Gut bacteria from MS patients influence T-cell differentiation, creating a proinflammatory environment and promoting T-cell-mediated autoimmunity.	↑ *Akkermansia muciniphila* and *Acinetobacter calcoaceticus*. ↓ *Parabacteroides distasonis*. Fecal transfer of MS-derived bacteria exacerbated experimental autoimmune encephalomyelitis symptoms in mice.	Established RR-MS	[100]
Observational/Intervention (Human/Animal)	MS Twins vs. Healthy Twins; Germ-Free Mice Genetically Susceptible to Experimental Autoimmune Encephalomyelitis	Fecal microbiota transplant from MS twins or healthy twins into germ-free mice. Immune cell analysis.	The MS-associated microbiota directly programs immune cells toward a proinflammatory state by reducing the production of the regulatory cytokine IL-10.	Fecal microbiota transplant from MS twins induced a significantly higher incidence of EAE. Immune cells of mice receiving MS samples produced lower amounts of IL-10. Neutralization of IL-10 in healthy-colonized mice increased experimental autoimmune encephalomyelitis incidence.	Established MS (Causal Implication)	[103]

α-syn: Alpha-Synuclein; Aβ: Amyloid-β; AD: Alzheimer’s Disease; APP: Amyloid Precursor Protein; CNS: Central Nervous System; IL-10: Interleukin-10; IL-17: Interleukin-17; IL-22: Interleukin-22; iRBD: Idiopathic Rapid Eye Movement Sleep Behavior Disorder; IVIg: Intravenous Immunoglobulin; LPS: Lipopolysaccharide; MCI: Mild Cognitive Impairment; MS: Multiple Sclerosis; PD: Parkinson’s Disease; RR-MS: Relapsing–Remitting Multiple Sclerosis; SCFA: Short-Chain Fatty Acid; Th: T Helper cells; Th1/Th17/Th22: T Helper Subsets. ↑: increase; ↓: decrease.

**Table 2:** Overview of neurotropic viruses: genomic characteristics, CNS entry mechanisms, and their roles in neurodegenerative pathogenesis.

Genome	Virus	Viral Family	Target Cells (CNS/Periphery)	CNS Entry Mechanism	Role in Neurodegeneration	Correlation with Neurodegenerative Diseases/Pathologies	Ref.
DNA (dsDNA)	HSV-1	Herpesviridae	Neurons (trigeminal/olfactory ganglia), glia	Retrograde axonal transport (olfactory/trigeminal)	Promotes Aβ plaque formation and tau hyperphosphorylation	AD (Aβ deposition, tau hyperphosphorylation), Encephalitis	[109,110,111,112,114,115]
DNA (dsDNA)	HSV-2	Herpesviridae	Peripheral neurons	Axonal transport	Induces chronic neuroinflammation and neuronal stress	Meningitis	[113]
DNA (dsDNA)	VZV-2	Herpesviridae	Neurons (dorsal root ganglia), satellite cells	Axonal transport/hematogenous	Leads to vasculopathy and indirect reactivation of other latent viruses	Cognitive decline, AD	[116,117]
DNA (dsDNA)	EBV	Herpesviridae	B lymphocytes, epithelial cells	Indirect (immune-mediated, molecular mimicry)	Molecular mimicry triggers autoimmune attacks on myelin	MS (molecular mimicry between EBNA1 and myelin basic protein)	[118,119]
Retrovirus (ssRNA-RT)	HIV-1	Retroviridae	CD4^+^ T cells, microglia, macrophages	Hematogenous (early BBB crossing, “Trojan horse” mechanism)	Induces excitotoxicity, oxidative stress, and synaptic loss	HIV-associated neurocognitive disorders	[120]
Retrovirus (ssRNA-RT)	HTLV-1	Retroviridae	CD4^+^ T cells	Infiltration of infected T cells (paracellular entry), virological synapse	MMP-driven BBB disruption and motor neuron damage	HTLV-1-associated myelopathy/tropical spastic paraparesis; Spinal cord motor neuron damage	[121,122]
RNA (ssRNA+)	West Nile Virus	Flaviviridae	Neurons	BBB disruption/hematogenous spread	Upregulates α-syn expression in primary neurons	Parkinsonian symptoms (increased α-syn expression)	[123,124]
RNA (ssRNA+)	Japanese Encephalitis Virus	Flaviviridae	Dopaminergic neurons	BBB disruption/hematogenous spread	Damage to areas rich in dopaminergic neurons; catecholamine decline	Parkinsonian symptoms (decline in catecholamines/dopamine)	[125]
RNA (ssRNA+)	Dengue Virus	Flaviviridae	Neurons	BBB disruption/hematogenous spread	Increases risk of acute and long-term motor/cognitive impairment	Parkinsonian symptoms	[126,127,128]
RNA (ssRNA+)	SARS-CoV-2	Coronaviridae	Neurons, glia (ACE2 receptor), endothelial cells	Olfactory pathway, infected immune/endothelial cells	Accelerates α-syn aggregation and Lewy body formation	Parkinsonian symptoms (α-syn aggregation), Cortical atrophy, White matter damage	[129,130,131]

α-syn: Alpha-synuclein; Aβ: Amyloid-β; ACE2: Angiotensin-converting enzyme 2; AD: Alzheimer’s disease; BBB: Blood–brain barrier; CNS: Central nervous system; dsDNA: Double-stranded DNA; EBV: Epstein–Barr virus; HIV-1: Human immunodeficiency virus type 1; HSV-1/2: Herpes simplex virus type 1/2; HTLV-1: Human T-lymphotropic virus type I; MMP: Matrix metalloproteinase; SARS-CoV-2: Severe acute respiratory syndrome coronavirus 2; ssRNA: Single-stranded RNA; VZV: Varicella-zoster virus.

**Table 3:** Molecular mechanisms of neuroinvasion: viral receptors and CNS spread pathways.

Viral Family	Example Virus	Primary Host Receptor(s)	Mechanism of CNS Spread
Alphaviridae	Chikungunya Virus, Venezuelan Equine Encephalitis Virus	Matrix Remodeling-Associated 8, Low-Density Lipoprotein Receptor Class A Domain-Containing 3	Olfactory neuroepithelium; Active axonal transport
Rhabdoviridae	Rabies Virus	Nicotinic Acetylcholine Receptor, Neural Cell Adhesion Molecule	Retrograde axonal transport via motor neurons
Herpesviridae	HSV-1, HSV-2, VZV	Nectin-1, Herpesvirus Entry Mediator	Latency in sensory ganglia; Axonal spread
Coronaviridae	SARS-CoV-2	Angiotensin-Converting Enzyme 2, Neuropilin-1	Olfactory bulb pathway; BBB disruption
Flaviviridae	West Nile Virus, Japanese Encephalitis Virus	T-cell Immunoglobulin and Mucin-Domain, Dendritic Cell-Specific Intercellular Adhesion Molecule-3-Grabbing Non-Integrin	“Trojan Horse” mechanism (infected monocytes); Transcytosis

BBB: Blood–brain barrier; HSV-1/2: Herpes simplex virus type 1/2; SARS-CoV-2: Severe acute respiratory syndrome coronavirus 2; VZV: Varicella-zoster virus.

## Data Availability

No new data were created or analyzed in this study. Data sharing is not applicable to this article.

## Abbreviations

The following abbreviations are used in this manuscript:

AD	Alzheimer’s disease
Aβ	β-amyloid
α-syn	Alpha-synuclein
ALS	Amyotrophic lateral sclerosis
ANS	Autonomic nervous system
APP	Amyloid precursor protein
BBB	Blood–brain barrier
CNS	Central nervous system
DAMPs	Damage-associated molecular patterns
EBV	Epstein–Barr virus
ENS	Enteric nervous system
GABA	Gamma-aminobutyric acid
IFN-gamma	Interferon-gamma
IL	Interleukin
iRBD	Idiopathic rapid eye movement sleep behavior disorder
IPA	Indolepropionic acid
IVIg	Intravenous immunoglobulin
HBV	Hepatitis B virus
HCV	Hepatitis C virus
HFMD	Hand, foot, and mouth disease
HGT	Horizontal gene transfer
HPA	Hypothalamic–pituitary–adrenal axis
HSV	Herpes simplex virus
LPS	Lipopolysaccharide
MBP	Myelin basic protein
MCI	Mild cognitive impairment
MS	Multiple sclerosis
NDDs	Neurodegenerative disorders
PAMPs	Pathogen-associated molecular patterns
PD	Parkinson’s disease
ROS	Reactive oxygen species
RNS	Reactive nitrogen species
RR-MS	Relapsing–remitting multiple sclerosis
RSV	Respiratory syncytial virus
SARS-CoV-2	Severe acute respiratory syndrome coronavirus 2
SCFAs	Short-chain fatty acids
TLR3	Toll-like receptor 3
Th	T helper cells
Treg	Regulatory T cells
